# GhostConv+CA-YOLOv8n: a lightweight network for rice pest detection based on the aggregation of low-level features in real-world complex backgrounds

**DOI:** 10.3389/fpls.2025.1620339

**Published:** 2025-08-13

**Authors:** Fei Li, Yang Lu, Qiang Ma, Shuxin Yin, Rui Zhao

**Affiliations:** College of Information and Electrical Engineering, Heilongjiang Bayi Agricultural University, Daqing, China

**Keywords:** rice pest detection, GhostConv, context aggregation block, Shape-IoU, Slide Loss

## Abstract

Deep learning models for rice pest detection often face performance degradation in real-world field environments due to complex backgrounds and limited computational resources. Existing approaches suffer from two critical limitations: (1) inadequate feature representation under occlusion and scale variations, and (2) excessive computational costs for edge deployment. To overcome these limitations, this paper introduces GhostConv+CA-YOLOv8n, a lightweight object detection framework was proposed, which incorporates several innovative features: GhostConv replaces standard convolutional operations with computationally efficient ghost modules in the YOLOv8n’s backbone structure, reducing parameters by 40,458 while maintaining feature richness; a Context Aggregation (CA) module is applied after the large and medium-sized feature maps were output by the YOLOv8n’s neck structure. This module enhance low-level feature representation by fusing global and local context, which is particularly effective for detecting occluded pests in complex environments; Shape-IoU, which improves bounding box regression by accounting for target morphology, and Slide Loss, which addresses class imbalance by dynamically adjusting sample weighting during training were employed. Comprehensive evaluations on the Ricepest15 dataset, GhostConv+CA-YOLOv8n achieves 89.959% precision and 82.258% recall with improvements of 3.657% and 11.59%, and the model parameter reduced 1.34%, over the YOLOv8n baseline while maintaining a high mAP (94.527% vs. 84.994% baseline). Furthermore, the model shows strong generalization, achieving a 4.49%, 5.452%, and 3.407% improvement in F1-score, precision, and recall on the IP102 benchmark. This study bridges the gap between accuracy and efficiency for in field pest detection, providing a practical solution for real-time rice monitoring in smart agriculture systems.

## 1 Introduction

Heilongjiang province, situated in northeastern China (121°11^′^–135°05^′^ *E*, 43°26^′^–53°33^′^ *N*), with good water and heat conditions, providing a favorable climate for rice growth (Gao and Liu, 2011). However, rice insect pests will cause considerable yield losses yearly, threatening China’s ‘rice bowl.’ Pests and diseases not only affect rice production but also cause ecological damage due to the use of pesticides (Dawei et al., 2019). Therefore, timely and rapid identification and detection of rice insect pests have become necessary for agricultural production and environmental protection.

Traditional methods of identifying rice pests mainly rely on manual identification. These methods have the shortcomings of intense subjectivity, low efficiency, time-consuming, and labor-intensive (Zhao et al., 2021). Machine learning-based pest and disease detection methods use SIFT (Dawei et al., 2019), HOG, LBP, etc., to extract shape, color, and texture features. Then it uses SVM (Yalcin and Razavi, 2016), backpropagation (BP) (Wang et al., 2012; Zhao et al., 2008) neural network, Bayesian (Sachdeva et al., 2021) to classify the image into different categories according to the extracted features. However, the above method can only achieve the best effect in high-quality images with high contrast between insect and non-pest areas and low image noise. Meanwhile, changing the threshold or redesigning the algorithm is often necessary when the imaging environment or plant pest class changes (Wang et al., 2025). Significantly, the above method is lacking in the recognition effect in a complex environment where crops grow naturally (Zhang et al., 2022).

Many rice conditions exhibit unique visual symptoms well-suited for deep learning-based identification and classification. For example, (Rajalakshmi et al., 2021) focused on pest monitoring using a dataset comprising 16 categories of pests captured in the field through camera imagery. They then employed CNN to develop a robust framework capable of accurately identifying and classifying the pest species. (Azath et al., 2021) also use CNN to detect common cotton leaf diseases and pests. (Tetila et al., 2020) used UAVs equipped with high-resolution cameras to obtain soybean pest images and deployed various deep-learning models, including VGG, CNN, YOLO series, etc. These models analyzed the collected soybean pest images, aiming to identify and classify pests that commonly infest soybean plants. (Singh et al., 2021) used multiple image segmentation methods to automatically segment the pest-infected areas of the collected coconut palm images. Then, the pre-trained VGG, InceptionResNetV2, and DenseNet, *etc.* were fine-tuned through the inductive transfer method to classify coconut palm diseases or pests. In (Wang et al., 2021), the authors proposed improved YOLOv3 to detect tomato diseases and pests in a natural growth environment, which mainly involves three improvements: (1) Dilated convolutions are used instead of ordinary convolutions in YOLOv3’s backbone network, (2) in the detection head, the IOU threshold and linear attenuation confidence are employed to improve the accurate recognition of occluded pests, (3) the convolution decomposition and optimized loss function are used to make the model lightweight and fast. This method is suitable for rapid diagnosis of pests and diseases in a large number of video images.

Deep learning networks have been extensively applied to pest detection across various plant species using datasets collected under controlled laboratory conditions, achieving high detection accuracy (Rançon et al., 2019; Jiang et al., 2019; Xie et al., 2020). However, the complexity of natural environments, where plants grow, introduces significant challenges (Zhang et al., 2022). Pests have evolved to camouflage with their surroundings, often displaying body colors similar to the background, which complicates the deep learning model’s ability to extract low-level features from pest images accurately (Xu et al., 2022). Furthermore, external factors, such as camera shake, can lead to image blurring and ghosting, ultimately degrading the quality of the collected data. Additionally, obstacles like plant leaves and branches can obscure pests, and the wide variety of pest species further complicates detection (Javidan et al., 2024). These factors collectively impair the detection performance of deep learning architectures under naturalistic plant growth conditions. Despite deep learning techniques being widely applied for pest detection in crops like grains, tomatoes, and corn, and substantial work in the classification and detection of rice diseases (Hu et al., 2023; Guo et al., 2024; Uddin et al., 2024), research focusing specifically on rice pest detection remains scarce. This paper has made contributions in the following aspects:

1. A domain-specific dataset, RicePest15, has been developed to support multi-scale detection of rice pests, which annotated imagery with rectangular bounding boxes. First, the dataset comprises 1,712 images of rice plants both healthy and infected with 15 pests that were initially collected, encompassing diverse environmental variables such as illumination differences, shadow interference, and occlusion. Secondly, to generate instance-level annotations, the Segment Anything Model (SAM) was employed to extract segmentation masks corresponding to pest targets. Then, these masks were converted into rectangular bounding boxes, providing standardized annotation formats suitable for object detection models. Finally, to enhance dataset variability and model generalization, a suite of augmentation techniques encompassing geometric transformations (rotation, translation, scaling, and shearing), color space modifications (HSV adjustment), and compositional strategies (Mosaic augmentation) were applied. The RicePest15 dataset offers a comprehensive and field-realistic valid benchmark for the development and evaluation of automated pest detection algorithms in rice cultivation systems.
2. A lightweight model, GhostConv+CA-Yolov8n, was developed by incorporating several key innovations. Initially, GhostConv replaced conventional convolution operations in Yolov8n’s backbone to reduce the model’s parameter size. Then, after the neck network outputs large and medium-sized feature maps, a Context Aggregation (CA) module is introduced to enhance feature representation. This module aggregates the location information of low-level features with deeper semantic details, and it improves the model’s efficiency when the rice detection images contain fewer and larger pests. Finally, the model integrates Shape-IoU to emphasize the scale and shape of the rice pest bounding box, thereby enhancing pest boundary detection accuracy. To further address the issue of low classification accuracy due to category imbalance among samples, Slide loss is employed to improve the model’s overall performance.
3. An ablation experiment and comparison were conducted on the GhostConv+CA-YOLOv8n model using the Ricepest15 dataset and compared with YOLOv3 – YOLOv10, SSD, and FasterRCNN models. The results indicated that GhostConv+CA-YOLOv8n reduced the number of parameters and gradients by 40,458 to the YOLOv8n. GhostConv+CA-YOLOv8n achieved precision, recall, F1, *mAP*_0_._5_, and *mAP*_0.5−0.9_ of 89.959%, 82.258%, 85.9363%, 94.527%, and 55.579%, respectively, outperforming all other models. Further experimental verification on the public dataset IP102 demonstrated improvements in precision, recall, F1 score, *mAP*_0_._5_, and *mAP*_0.5−0.9_ by 5.452%, 3.407%, 4.4912%, 0.352%, and 0.003%, respectively, compared to the traditional YOLOv8n model. The GhostConv+CA-YOLOv8n model proved capable of lighter, quickly, and effectively identifying common rice pests in natural field environments, offering significant assistance in crop protection for farmers.

## 2 Related work

The discussion begins with the various categories of rice pests based on their preferred feeding environments. Following this, the improved YOLOv8n architecture is examined in detail.

### 2.1 Categories of rice pests

**Figure 1:**
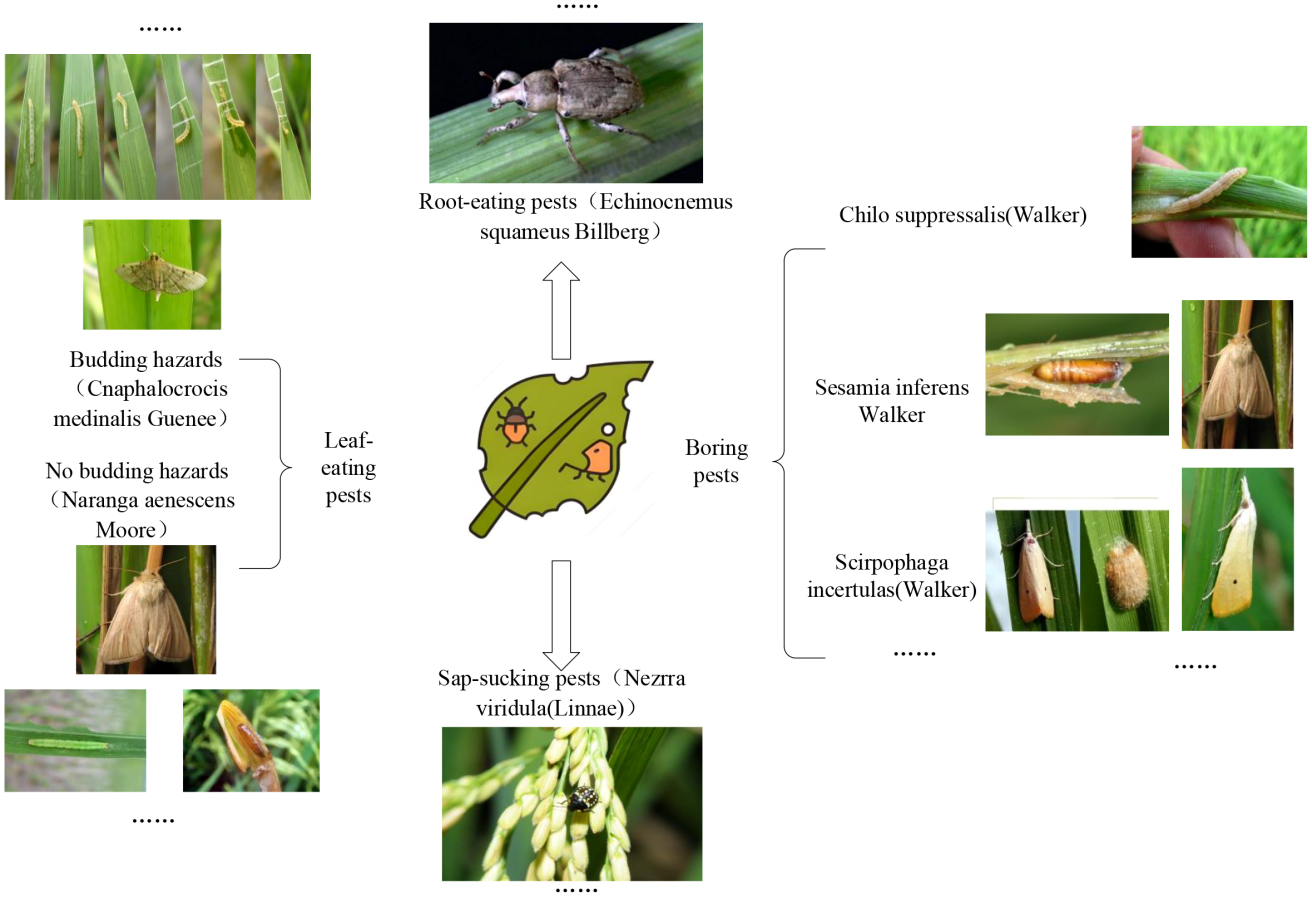
Categories of rice pests.

Rice plants were classified into three categories according to the preferred feeding sites of pest species (**Figure 1**).

The first type is common pests that mostly like to feed on leaves, such as Cnaphalocrocis Medinalis Guenee and Rice skipper (Parnara Guttata Bremeret Grey). They usually destroy rice throughout the year by infusing rice leaves into bracts by larvae spinning and feeding on the upper epidermis or young leaf sheath in the bracts. It causes the leaves to be incomplete, and in severe cases, only the midrib remains, causing the crops to grow slowly or even wither. In addition, other pests that like to eat rice leaves include the Rice Green Semilooper (Naranga Aenesc), Spodoptera Litura, Rice green horned caterpillar, Oxya chinensis, *etc.* Most of the rice caterpillars appear in the stems of rice; they are borers, such as Chilo Suppressalis (Walker), Scirpophaga incertulas (Walker), and Sesamia inferens (Walker). They cause dead heart seedlings, dry ears, and insect injuries during tillering. Most are in the third, fourth, and adult stages, with increased food intake and eating leaves, and have specific aggregation behaviors (shengguang, 2021).

The second is sap-sucking pests, including Rice planthopper (white-backed and black planthopper), Nephotettix Cincticeps, and Inazuma dorsal. They suck sap on rice leaves and leaf sheaths with adults and nymphs, causing the growth of the injured plants to be inhibited, causing the whole plant to turn yellow or wither, affecting crop yield and being difficult to control (shengguang, 2021).

The last one is root-eating pests, generally vulnerable in dry farming and sandy fields with good aeration and low water content. Pests such as Echinocnemus squamous Billberg, Rice water weevil, Donacia provost Fairmaire, etc., mainly harm the rice fibrous root with larvae, causing the injured rice to grow poorly, the leaves to be yellow, the plants to be short, and the profound rice roots to be bitten off or even die in pieces (Fu Qiang, 2019).

### 2.2 Improved YOLOv8n

YOLOv8 is a single-stage model that improves and innovates on the previous YOLOv5 and has a wide range of applications in the agricultural field. YOLOv8 is engineered for speed, accuracy, and simplicity. It has multiple variants, including YOLOv8n, YOLOv8s, YOLOv8m, YOLOv8l, and YOLOv8x architectures, each designed for different performance and computational efficiency trade-offs. YOLOv8n and YOLOv8s are more suitable for resource-constrained environments, YOLOv8n utilizes fewer parameters and less complex layers. YOLOv8m serves as a balanced option, offering a compromise between speed and accuracy, making it appropriate for moderately resource-limited settings. In contrast, YOLOv8l and YOLOv8x provide higher accuracy for more demanding detection tasks, albeit at the expense of increased computational resources (Sohan et al., 2024).

**Figure 2:**
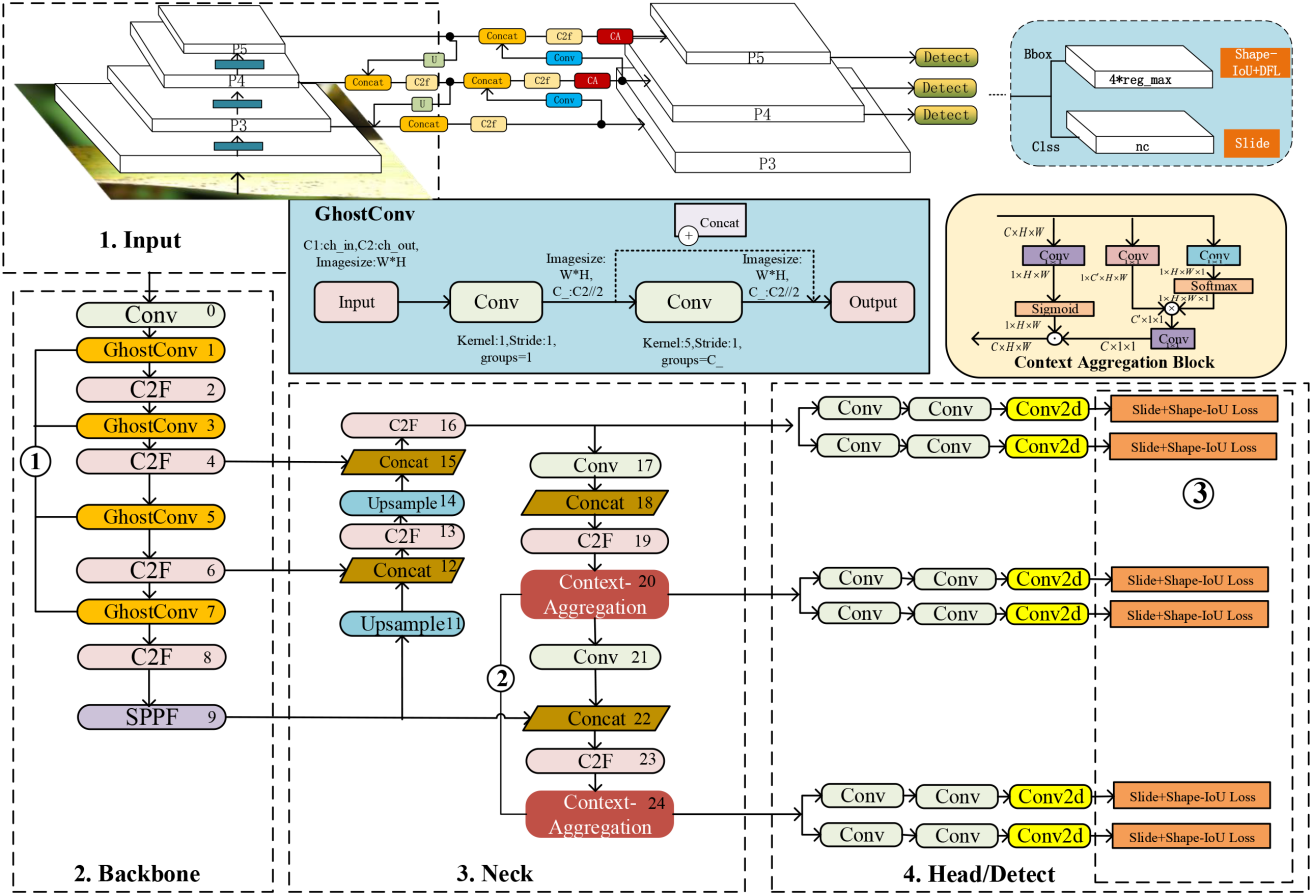
Architecture of GhostConv+CA-YOLOv8n.

This paper analyzes the nonlinear characteristics of the original collected pest images and the actual bounding box distribution of the target and enhances the original image to obtain Ricepest15. Building upon the YOLOv8n architecture as the foundational model, this study introduces several architectural modifications: the standard convolutional operations in YOLOv8n’s backbone are substituted with GhostConv modules to reduce computational complexity. Meanwhile, a Context Aggregation attention mechanism is integrated into the neck to improve feature representation. Furthermore, a composite loss function combining Shape-IoU and Slide loss is employed to optimize localization performance. The resulting model denoted GhostConv+CA-YOLOv8n, demonstrates improved efficiency and detection accuracy in pest recognition tasks. The GhostConv+CA-YOLOv8n’s architecture is shown in **Figure 2**. Among them, ①②③ are the specific parts to be improved. The 1 is input data, and the 2,3,4 network structure together constitutes YOLOv8n, and the details of the improvements are in Section 3.3.

In YOLOv8n, the input part mainly performs data augmentation (Mosaic) and preprocessing (Letterbox) on the graphics. By default, data Mosaic enhancement has not been performed in the last ten training rounds. The backbone network of YOLOv8n is composed of Conv, C2f, and SPPF(Spatial Pyramid Pooling–Fast) structures (He et al., 2015), which mainly refer to the ELAN structure of YOLOv7 (Wang et al., 2023b). The neck network adopts a path aggregation network (PAN) structure (Yin et al., 2025), which can enhance the network’s feature fusion capabilities for objects of different scales. The prediction head adopts a decoupled structure and divides the tasks into category score prediction (Binary Cross Entropy, BCE) (Ruby et al., 2020), bounding box regression, and object probability, where the distribution focus loss (DFL) (Xie et al., 2024) and the complete intersection-over-union (CIOU) loss (Wang and Song, 2021) are used in the regression task. The aligned assigner allocation strategy used in the YOLOv8n network selected positive samples according to the score weighting results of classification and regression. The purpose of C2f is to reduce parameters while retaining rich gradient flows. Bottleneck is inspired by the residual module of Darknet. It consists of two convolution modules and shortcuts. The convolution is processed by Conv2d, BatchNorm, and the activation function (LeakyReLU) (Xu et al., 2020), and the convolution kernel size is 3 × 3. The first convolution of Bottleneck reduces the number of input channels to 0.5 times the number of intermediate channels, and then the second convolution restores the channels to the number of output channels. When the number of input and output channels is the same and shortcut=True, the original input features are added with the extracted features to maintain the feature dimension unchanged. This design structure can retain and fully utilize the input features and alleviate the gradient disappearance problem (Zhang et al., 2023).

### 2.3 Pest detection methods

Plant pest detection methods are divided into two-stage and single-stage networks, representative models including FasterRCNN Ren et al. (2017) and YOLO, SSD Redmon et al. (2016); Redmon and Farhadi (2017, 2018). For example, Uygun and Ozguven (2024) are committed to advancing the segmentation of multiple pests, specifically targeting Tuta Absoluta pests on tomato plant leaves, using the YOLOv8l-Seg model and achieving *mAP*_0_._5_, *mAP*_0.5−0.95_, precision and recall rates of 93.5%, 80.%6, 95.6% and 85.9% respectively when the input image size is 640x640. Fuentes et al. (2017) focused on detecting pests and diseases in tomatoes using camera images. Their study compared several models (FasterRCNN, R-FCN, and SSD) on this task. The results showed that R-FCN using ResNet-50 surpassed all other models with an *mAP* of 85.98%. Single-stage deep learning models, such as YOLO Islam et al. (2023) and SSD Adem et al. (2023), have faster inference capabilities Hu et al. (2024); Meng et al. (2023); Sampurno et al. (2024); Wang et al. (2023a), and can be applied to real-time tasks in the agricultural field.

In addition to the studies mentioned, recent advancements in rice disease and pest detection utilizing two-stage and single-stage deep learning models are summarized in **Table 1**.

**Table 1:** Summary of two-stage and single-stage based rice diseases and pests detection.

Method	Paper	Crop	Objective	Dataset	Models	Performance
two-stage	(Liu et al., 2023)	Rice canopy	Pests detection and segmentation	Multisource data sets(8018)	GA-Mask R-CNN	mAP–92.71%,recall–89.28%,F1–90.96%.
	(Zhou et al., 2019)	Rice	Diseases detection	3010 images	FCM-KM, FasterRCNN	Accuracy and time of rice blast–96.71%/0.65s, bacterial blight–97.53%/0.82s, blight–98.26%/0.53s.
	(Bari et al., 2021)	Rice leaf	Diseases detection	RLDD (2400)	Faster R- CNN	Accuracy of rice blast– 98.09%, brown spot– 98.85%, and hispa–99.17%,healthy– 99.25%.
Single-stage	(Chen et al., 2022)	Rice granary	Pest detection	Take photos	YOLOv4	*mAP*–97.55%
	(Yin et al., 2024)	Rice	Pest detection	The camera captured images(2500)	Improved YOLOv8	Average precision– 95.8%, F1-score– 94.6%.
	(Jain et al., 2022)	Rice	Disease detection and classification	Images(762)	YOLOv3 tiny and YOLOv4 tiny	mAP of YOLOv4 tiny–97.36%, YOLOv3 tiny– 79.77%.
	(Kumar et al., 2023)	Rice leaf	Disease detection	The Kaggle rice leaf disease (RLD)(850)	YOLOv5	*mAP*0.5– 70.8%,precision– 82.8%,recall– 75.1%.
	(Chen et al., 2025)	Rice	Rice diseases and pests detection	Images From the AR glasses (3856)	Improved YOLOv5X model with AF-FPN	mAP– 87.4%,precision– 82.3%,recall– 86.5%.

Moreover, the study by (Liu et al., 2025) focuses on identifying small rice pests using datasets such as IP102 (2316 images), PaddlePaddleAIStudio (2465 images), and mobile phone camera captures (2393 images). Their proposed RP-DETR model achieves a *mAP*_0_._5_ of 92.2%, precision of 91.9%, and recall of 85.8%. On the self-constructed RicePest18 dataset, it attains an 80.4% F1-score, 87.4% *mAP*_0_._5_, and 28.7% *mAP_s_* for small targets, showing improvements of 5.3% and 5.1% over the baseline. In contrast, (Chen et al., 2020) addresses rice disease detection using a dataset of 500 rice plant disease images, employing a DenseNet model pre-trained on ImageNet with an Inception module, achieving an average accuracy of 98.63%.

In summary, numerous advanced models have been utilized for crop pest and disease detection, classification, and segmentation. Typically, models that prioritize detection accuracy are designed with a two-stage architecture, while those focused on detection speed are often based on a single-stage structure. Furthermore, these models have been applied to cotton (Tetila et al., 2020), grapes (Sanath Rao et al., 2021), sugar beets (Ozguven and Adem, 2019), and tomatoes (Uygun and Ozguven, 2024), with detection *mAP*_0_._5_ and accuracy achieving high values across these crops. Moreover, the detection and classification of rice leaf diseases and pests have progressed, with accuracy exceeding 70%. Above studies (**Table 1**) demonstrate advancements in rice pest and disease detection using deep learning approaches. However, rice pest detection methods based on deep learning networks typically require extensive image datasets for training to ensure model generalizability. Furthermore, deep learning models rely on millions of built-in parameters for classification and pest localization. During the implementation and deployment phases, a trade-off between computational complexity and accuracy is often necessary.

## 3 Materials and methods

### 3.1 Experimental materials

Images were captured from pest occurrences on various rice cultivars grown in the experimental fields of Heilongjiang Bayi Agricultural University, China. The field coordinates are as follows: 125°10^′^49.730^′′^ *E*,46°35^′^25.383^′′^ *N* to 125°10^′^51.412^′′^ *E*,46°35^′^23.421^′′^ *N*, covering an area of approximately (31 × 112)m^2^+(88 × 14)m^2^ = 4704m^2^. Rice pest images were captured between June 13, 2023, and September 30, 2023, as well as July 13, 2024, and August 31, 2024.

Furthermore, image acquisition was performed using a mobile phone device (Redmi Note 11T Pro, Xiaomi, China) with three rear cameras: Rear camera 1 is the main camera with 64 million pixels. Rear camera 2 (8 million pixels) is an ultra-wide-angle lens for capturing macro and large-scene images. The rear camera 3 (2 million pixels) is a macro lens that captures micro and detailed photos. Among them, the wide-angle shooting angle is 120°, supporting rear video shooting (4K@30 frames*/*second, 1080p@30*/*60 frames*/*second 720p*/*30 frames*/*second), video recording (1080p*/*120fps 720p@120*/*240*/*960fps) and slow-motion video recording. The images of rice leaves and stems taken by the mobile phone camera come from a rice field with an area of about 4704 square meters.

#### 3.1.1 Data collection and data labeling methods

This study utilizes color images of both healthy and insect-infested rice plants as research subjects. Field photography and online collection methods were employed for image acquisition. During field photography, the camera was positioned at a distance of 10*cm* to 18*cm* from the target to ensure that the rice plants were centrally aligned within the frame.

The camera’s focal length and aperture were automatically set, along with automatic white balance. Image collection was conducted daily between 15:00 and 16:00, with the resolution set to 2,000 × 1,325 pixels. A total of 1712 original rice pest images were captured, showcasing both healthy plants and those with pest damage, under various lighting conditions and complex backgrounds.

**Figure 3:**
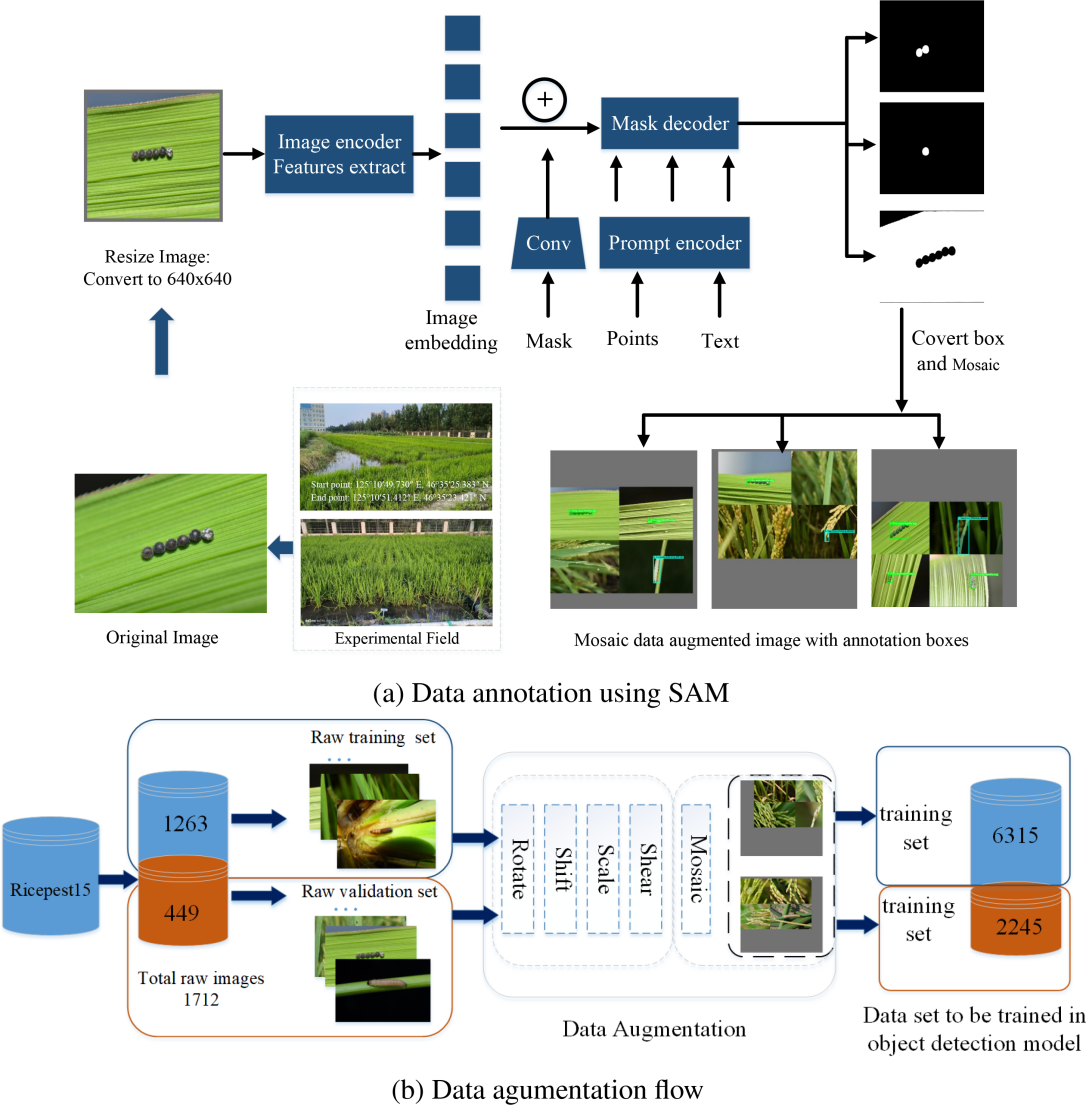
Data annotation and augmentation architecture.

In addition, based on the primary color map from ‘Diagnosis and Control of Rice Diseases, Pests, and Weeds’, the image data were labeled and proofread to construct the original rice pest dataset. Due to the labor-intensive and challenging process of regional box labeling required for object detection, the interactive data semantic annotation tool, SAM, was employed to facilitate automatic semantic recognition and annotation, thereby reducing the difficulty of manual data annotation, and it is a large segmentation model with strong zero-sample generalization ability, leverages a ViT image encoder to extract features from images. This architecture enables SAM to effectively handle segmentation tasks without task-specific training data. In SAM, the point or text is input processed to the prompt encoder, which is built into an embedding vector in real time. Then the mask decoder predicts the segmentation result and confidence score Kirillov et al. (2023). The interactive automatic annotation process for rice pests is illustrated in **Figure 3a**.

### 3.2 Data augmentation

The raw rice pest dataset was divided into training and validation subsets in an 80: 20 ratio. To address class imbalance and improve the model’s generalization capability, data augmentation methods were categorized into simple augmentation and Mosaic (**Figure 3b**). Simple augmentation techniques included rotating, shearing, shifting, scaling, and adjusting the HSV of images (Waheed et al., 2020). Mosaic (Bochkovskiy et al., 2020), a more advanced method, combines multiple images: Four training images are randomly selected, and simple augmentation operations are applied to each. These augmented images are then placed on a board of specified size, where the images are resized using LetterBox (Wang et al., 2023b) and arranged in a specific pattern, one on the left and one on the right in the first and second rows, forming a new image containing the target boxes. This method significantly enriches the background of the detected objects. Additionally, during the standardized calculation, data from all four images are processed simultaneously, improving both processing efficiency and the model’s robustness.

#### 3.2.1 Image rotation

Image rotation (Jain et al., 2022) can be analogized to the rotation of a point in the *XOY* coordinate plane. Specifically, a point *P* is *XOY* rotated counterclockwise by an angle *β* about the origin *O*, resulting in a new position at point *Q*.

Assuming point *P* has coordinates (*x,y*) and point *Q* has coordinates $\left(x^{\prime },y^{\prime }\right)$ in the *X,O,Y* coordinate system, and point *P* and the origin *O* distance is denoted as *D*, the rotation of point *P* by an angle *β* around the origin can be described using the following Equations 1, 2.


(1) $$\begin{array}{l} x^{\prime }=D\text{cos }(\alpha +\beta )\\ \;=D\left(\text{cos }\alpha \text{ cos }\beta -\text{sin }\alpha \text{ sin }\beta \right)\\ \;=x\text{ cos }\beta -y\text{ sin }\beta \end{array}$$

(2) $$\begin{array}{l} y^{\prime }=D\text{ sin }(\alpha +\beta )\\ \;=D\left(\text{cos }\alpha \;\text{sin }\beta +\text{sin }\alpha \;\text{cos }\beta \right)\\ \;=x\;\text{sin }\beta +y\;\text{cos }\beta \end{array}$$

In digital images, which are represented as two-dimensional arrays, the above formula can be transformed into a matrix operation as Equations 3, 4:


(3) $$R=\left[\begin{array}{ccc} \text{cos }\beta & \text{sin }\beta & 0\\ -\text{sin }\beta & \text{cos }\beta & 0\\ 0 & 0 & 1 \end{array}\right]$$

(4) $$\left[\begin{array}{ccc} x^{\prime } & y^{\prime } & 1 \end{array}\right]=\left[\begin{array}{ccc} x & y & 1 \end{array}\right]*R$$

#### 3.2.2 Image shearing

Shearing (Xing et al., 2019) is a geometric transformation that involves scaling the signed distance from each point of a figure to a straight line, which is parallel to a specific direction, according to a predetermined ratio. This operation is typically performed in the *X* or *Y* -direction. Shearing along *Y* -axis transformation can be achieved using the following Equations 5–7:


(5) $$x+\text{sin }\alpha *Y=x^{\prime }$$

(6) $$y*\text{cos }\alpha =y^{\prime }$$

(7) $$\left[\begin{array}{c} x^{\prime }\\ y^{\prime }\\ 1 \end{array}\right]=\left[\begin{array}{ccc} 1 & \text{sin }\alpha & 0\\ 0 & \text{cos }\alpha & 0\\ 0 & 0 & 1 \end{array}\right]*\left[\begin{array}{c} x\\ y\\ 1 \end{array}\right]$$

#### 3.2.3 Image shifting

Image shifting (Xing et al., 2019) involves translating an image by a *x_t_* along the *X*-axis and *y_t_* along the *Y* -axis. This operation can be mathematically represented as Equations 8, 9:


(8) $$x^{\prime }=x+x_{t}$$

(9) $$y^{\prime }=y+y_{t}$$

Here, any point in the image is (x,y) (coordinates), which are then translated by $x_{t}$ and $y_{t}$ on the x and y axes, respectively, to obtain the new coordinates $\left(x^{\prime },y^{\prime }\right)$ where the point should be. The conversion into an image matrix is as Equation 10:


(10) $$\left[\begin{array}{c} x^{\prime }\\ y^{\prime }\\ 1 \end{array}\right]=\left[\begin{array}{ccc} 1 & 0 & x_{t}\\ 0 & 1 & y_{t}\\ 0 & 0 & 1 \end{array}\right]*\left[\begin{array}{c} x\\ y\\ 1 \end{array}\right]$$

#### 3.2.4 Image scaling

Image scaling (Xing et al., 2019) is performed by applying distinct scaling factors ( $x_{zoom}$ and $y_{zoom}$ ) for the *x* and *y* directions, to control the image’s shrinkage or enlargement in a specific proportion. This can be achieved through the following Equations 11–13:


(11) $$x^{\prime }=x\times x_{zoom}$$

(12) $$y^{\prime }=y\times y_{zoom}$$

(13) $$\left[\begin{array}{c} x^{\prime }\\ y^{\prime }\\ 1 \end{array}\right]=\left[\begin{array}{ccc} x_{zoom} & 0 & 0\\ 0 & y_{zoom} & 0\\ 0 & 0 & 1 \end{array}\right]*\left[\begin{array}{c} x\\ y\\ 1 \end{array}\right]$$

#### 3.2.5 HSV

HSV (Hue, Saturation, Value) (Liu et al., 2023) is based on the Hexcone Model and is commonly used for generating 8-bit and 16-bit images. In these images, the Red (R), Green (G), and Blue (B) channels are first converted to floating-point format and then scaled to fit within the range of 0 to 1. This transformation is performed using the following Equations 14, 15:


(14) V←max (Red,Green,Blue)

(15) $$H\leftarrow \left\{ \begin{array}{l} \frac{60\times \left(Green-Blue\right)}{V-min\left(Red,Green,Blue\right)}\;if\;\left(V=Red\right)\\ \frac{120+60\times \left(Blue-Red\right)}{V-min\left(Red,Green,Blue\right)}\;if\;\left(V=Green\right)\\ \frac{240+60\times \left(Red-Green\right)}{V-min\left(Red,Green,Blue\right)}\;if\;\left(V=Blue\right)\\ \begin{array}{ll} 0 & if\left(Red=Green=Blue\right) \end{array} \end{array}\right.$$

If H<0 , then the hue value is adjusted by adding 360 to it Equation 16:


(16) H←H+360

After this adjustment, the output values are constrained as Equation 17:


(17) 0≤V≤1,0≤S≤1,0≤H≤360

This ensures that the hue (*H*), saturation (*S*), and value (*V)* are within the appropriate ranges for proper representation in the HSV color model.

#### 3.2.6 Letterbox

The Letterbox operation (Wang et al., 2023b) is an image preprocessing technique designed to adjust the image size while preserving the original aspect ratio. This approach utilizes adaptive scaling technology to minimize information loss and geometric distortion, and the calculation process is as follows:

• The scaling ratio is first calculated by dividing the required size by the original image’s length (*L*_orig_) and width (*W*_orig_), to get two scaling ratios *S_L_* and *S_W_*. Then, the larger is selected as the scaling ratio;

• The scaled length *L*_scaled_ and scaled width *W*_scaled_ are given by Equations 18, 19:


(18) $$L_{\text{scaled}}=L_{\text{orig}}\times S_{L}$$

(19) $$W_{\text{scaled}}=W_{\text{orig}}\times S_{W}$$

Thus, the new dimensions of the scaled image are determined by multiplying the original dimensions by their corresponding scaling ratios.

• In YOLOv8n, if the ‘auto’ is set to True, the image will use padding to adjust the width and height by a multiple of the step size; if the ‘scale_fill’ parameter is set to True (this article uses this method), the image will be scaled according to the size of the target and padding will not work.

A dataset for rice pest detection, named Ricepest15, was constructed through data augmentation. The specific distribution of rice pests is presented in **Table 2**.

**Table 2:** Distribution of labeled and enhanced data for each type of rice pest.

Classes ID	Labeling objects	Classes name	Augmentation
0	714	Chilo_suppressalis_egg	4284
1	797	Chilo_suppressalis_Walker	4782
2	31	Chilo_suppressalis_pupa	186
3	23	Sesamia _inferens_adult	138
4	16	Erthesina_fullo_Thunberg_first_instar	96
5	321	Nezara_viridula_Nymph_three_instar	1926
6	51	Nezara_viridula_Nymph_fourth_instar	306
7	258	Nezara_viridula_Nymph_fifth_instar	1548
8	306	Nezara_viridula_adult	1836
9	280	Sesamia_inferens_Walker	1680
10	462	Naranga_aenescens_Moore_Walker	2772
11	145	Naranga_aenescens_Moore_adult	870
12	29	Cnaphalocrocis_medinalis_Guenee_Walker	174
13	3	Cnaphalocrocis_medinalis_Guenee_pupa	18
14	13	Sesamia_inferens_pupa	78

### 3.3 GhostConv+CA-YOLOv8n

#### 3.3.1 GhostConv

Due to constraints in memory and computing resources, ordinary convolution in YOLOv8n was replaced with GhostConv. The proposed GhostConv module implements parameter-efficient feature extraction through a dual-branch architecture: (1) Primary branch: Standard 3 × 3 convolutions capture spatial hierarchies; (2) Ghost branch: Depthwise separable convolutions generate complementary feature maps via cheap linear operations (*ϕ*), achieving 6.29% parameters reduction in ablation studies. GhostConv enhances the extraction of inherent feature information through multiple linear transformations and offers flexibility for integration into other models.

In YOLOv8n, the input is *X* ∈ R*^c^* ^×^ *^h^* ^×^ *^w^*, where *c*, *h*, and *w* are the channels and image sizes of the input image, respectively. The operation of generating *n* feature maps by a convolutional layer can be expressed as (Equation 20):


(20) Z=X∗f+bias

where $Z\in {\mathbb{R}}^{w^{\prime }\times h^{\prime }\times n}$ is the feature map with n channels through convolution ( * ), and $f\in {\mathbb{R}}^{c\times k\times k\times n}$ denotes the filters in this layer. $w^{\prime }$ , $h^{\prime }$ and k×k are output feature size and convolution kernel, respectively. In convolution, the number of parameters involved in the operation is n·c·k·k . Generally, n and c are very large (for example, 256 or 512), so the convolution parameters are often thousands in f and b .

In addition, the output feature maps are considered ‘ghosts’ and are derived from a few intrinsic feature maps through simple transformations. These intrinsic maps, typically smaller, are produced by standard convolution filters. Where $Z^{\prime }\in {\text{R}}^{w^{\prime }\times h^{\prime }\times m}$ ( m≤n ) is an intrinsic feature map generated by Equation 21.


(21) $$Z^{\prime }=X*f^{\prime }$$

where $f^{\prime }\in R^{c\times k\times k\times m}$ represents the filter used, and the bias term is omitted for simplicity. The hyperparameters, such as filter size, stride, and padding, match those in the standard convolution (Equation 20) to maintain the output feature map’s spatial size ( $w^{\prime }$ and $h^{\prime }$ ).

Linear operations are applied to the obtained intrinsic features ( $Z^{\prime }$ ) to derive s (ghost features) as described by Equation 22.


(22) $$z_{i,j}=\phi_{i,j}\left(z_{i}^{'}\right),\forall i=1,\ldots ,m,j=1,\ldots ,s$$

where $z_{i}^{'}$ represents the i -th intrinsic feature, while $\phi_{i,j}$ denotes the j -th linear operation (excluding the last one) that generates the j -th ghost feature map $z_{i,j}$ .

**Figure 4:**
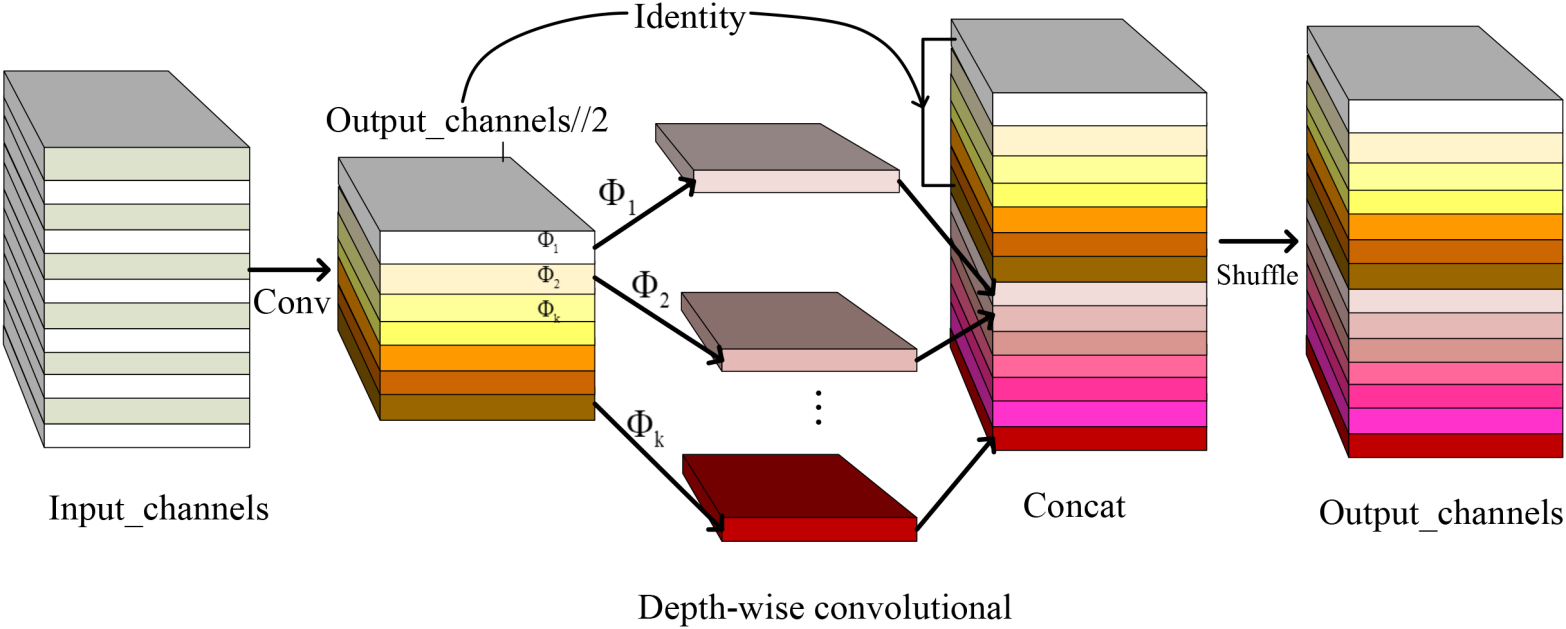
GhostConv module structure.

Consequently, each intrinsic feature map $z_{i}^{'}$ serves as the basis for generating ghost feature maps, denoted as ${\left\{z_{i,j}\right\}}_{j=1}^{s}$ , through inexpensive transformation operations. The final transformation $\phi_{i,s}$ represents an identity mapping, preserving the original intrinsic features, as illustrated in **Figure 4**. According to Equation 22, the output of the Ghost module consists of n=m·s feature maps, $Z=\left[z_{1,1},z_{1,2},\ldots ,z_{m,s}\right]$ . The linear operation ϕ is applied to each channel, requiring far less computation than standard convolution. GhostConv modules typically use convolution kernels of 3×3 and 5×5 to implement linear convolution.

The Ghost module, designed to reduce computational costs, operates similarly to an identity mapping and $m\cdot \left(s-1\right)=\frac{n}{s}\cdot \left(s-1\right)$ lightweight linear operations (depth-wise convolution). Each operation utilizes a convolutional kernel ofaverage size d×d (e.g., 3×3 or 5×5 ). The speedup ratio between the Ghost module and standard convolution was calculated, yielding the following results (using Equation 23).


(23) $$\begin{array}{l} r_{s}=\frac{n\cdot h^{\prime }\cdot w^{\prime }\cdot c\cdot k\cdot k}{\frac{n}{s}\cdot h^{\prime }\cdot w^{\prime }\cdot c\cdot k\cdot k+\left(s-1\right)\cdot \frac{n}{s}\cdot h^{\prime }\cdot w^{\prime }\cdot d\cdot d}\\ =\frac{c\cdot k\cdot k}{\frac{1}{s}\cdot c\cdot k\cdot k+\frac{s-1}{s}\cdot d\cdot d}\approx \frac{s\cdot c}{s+c-1}\approx s \end{array}$$

where the size of d×d , which is comparable to the size of k×k , and *s* ≪ *c*. Furthermore, the compression ratio of the model parameters is calculated using Equation 24.


(24) $$r_{c}=\frac{n\cdot c\cdot k\cdot k}{\frac{n}{s}\cdot c\cdot k\cdot k+\frac{\left(s-1\right)\cdot n}{s}\cdot d\cdot d}\approx \frac{s\cdot c}{s+c-1}\approx s$$

The compression and speed-up ratios of GhostConv and ordinary convolution are comparable both *s* times, indicating that GhostConv is lighter and runs faster.

The improved backbone structure can be divided into 10 parts (0-9). Layer 0 uses ^1^ 1.3.2 Context Aggregation Block In field-acquired pest images captured under natural growth conditions, scenarios involving fewer but larger-sized pests tend to benefit more from low-level visual cues. In such cases, features such as distinct edges, color contrasts, and texture patterns provide critical information that enhances the effectiveness of model training. While high-level features carry more significant semantic information, they suffer from low. × 3 ordinary convolution to extract low-level features. The standard convolutions in layers 1,3,5*,and*7 are replaced with GhostConv to lighten YOLOv8n’s backbone network, and they are responsible for capturing image lower characteristics, such as edges and textures, which are essential for the network’s feature extraction process. From **Figure 4**, all GhostConvs first use ordinary convolutions with a convolution kernel of 1 × 1, stride=1, groups=1, and the output feature map size remains unchanged. Then, the above ordinary convolution features are subjected to cheap linear convolution (Depth-wise convolutional) one by one. The convolution parameters are 5 × 5, *stride* = 1, *groups* = 7, and the activation is the SiLU function; Shuffle is used to generate *Output_channels//*2 ‘ghost’ features. The last (10-th) layer is SPPF, and this layer performs pooling operations with four kernel sizes: 1×1, 2×2, 3×3, and 6×6 on the input feature maps. The SPPF layer can capture objects of different sizes while preserving high-level semantic information within the feature maps. This multi-scale pooling method provides a processing solution for accurately understanding and identifying targets of various scales.

#### 3.3.2 Context aggregation block

**Figure 5:**
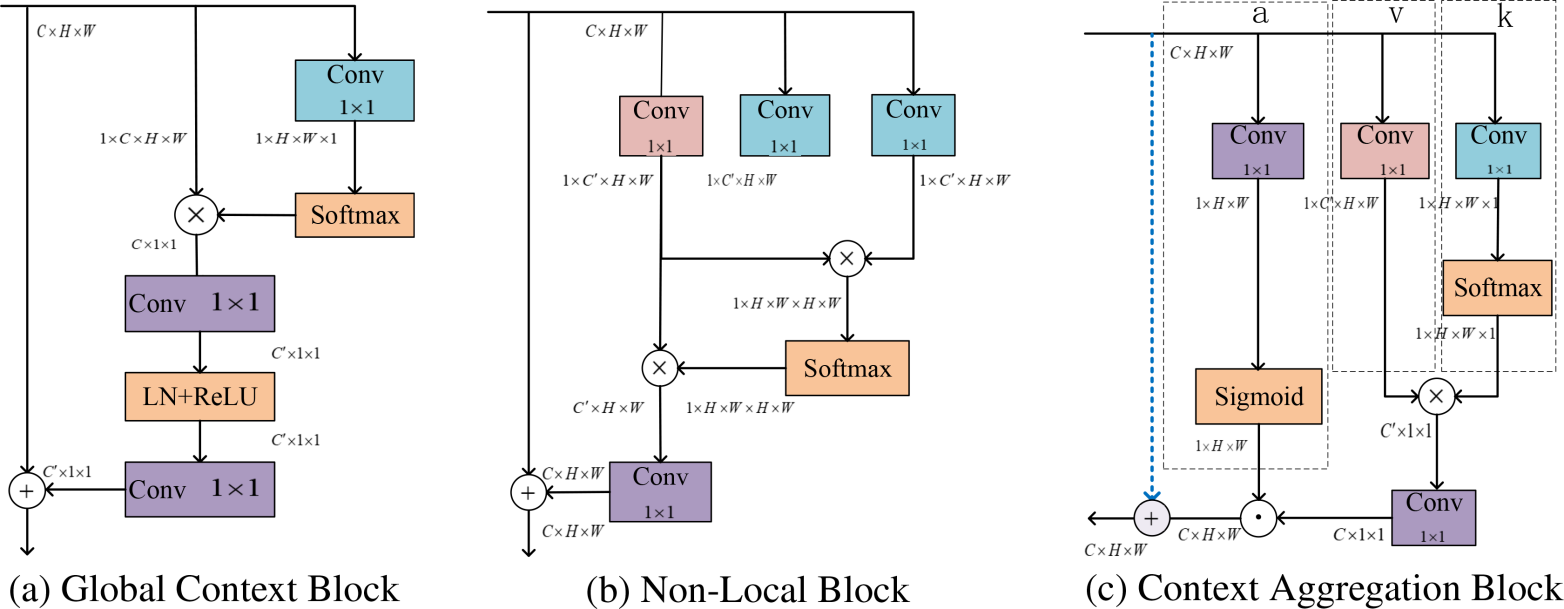
The rearrangement of feature maps, denoted by their dimensions like *C* × *H* × *W*, indicates a matrix with *C* channels, height *H*, and width *W*. ⊗ denotes the multiple matrix multiplications simultaneously, ⊙ refers to the element-wise multiplication between matrices, with broadcasting applied to handle shape mismatches, ⊕ indicating that matrices of different shapes are added element by element through broadcasting. The figure’s blue, red, and purple represent the attention map, feature mapping, and context-optimized convolutional layer operations, respectively.

In field-acquired pest images captured under natural growth conditions, scenarios involving fewer but larger-sized pests tend to benefit more from low-level visual cues. In such cases, features such as distinct edges, color contrasts, and texture patterns provide critical information that enhances the effectiveness of model training. While high-level features carry more significant semantic information, they suffer from low resolution and poor overall detail perception. To address this, after extracting the large and medium-sized feature maps from the neck, a Context Aggregation block (CA-block, **Figure 5c**) is incorporated. This block enhances the positional information of low-level features while simultaneously fusing the semantic information from high-level features, thereby improving the recognition accuracy of rice pests.

where GCNet Cao et al. (2023) (**Figure 5a**) is a simple but effective approach that combines the NonLocal Neural Network (NLNet) Wang et al. (2018) (**Figure 5b**) and the Squeeze and Excitation Network (SENet) Hu et al. (2018) into a lightweight module. Some of the architectures of these blocks are shown in **Figure 5**.

A context aggregation (CA) block is incorporated into the neck of the network, which captures pixel-level contextual information by aggregating spatial cues, formulated as Equation 25:


(25) $$Q^{j}=P^{j}+\sum_{j=1}^{N}[\frac{\exp\;(w_{k}p^{j})}{\sum_{m=1}^{N}\text{exp }(w_{k}p^{m})}\cdot w_{v}p^{j}]$$

where P and Q denote the input and output feature map, respectively, each comprising N pixels, and j,m∈{1,N} and N=H×W . The linear projection matrices $w_{k}$ and $w_{v}$ typically implemented via 1×1 convolutions layers. The above formula simplifies the self-attention mechanism and reduces the model parameters by replacing the matrix multiplication between query and key with linear transformation.

Moreover, a reweighting matrix, denoted as a , matching the shape of P and Q , is employed to adjust the global spatial context aggregation for each pixel as Equation 26.


(26) $$a^{j}=\frac{\text{exp }(w_{a}P^{j})}{\sum_{n=1}^{N}\exp\;(w_{a}P^{n})}$$

where j,n={1,N} are the matrix indices.

#### 3.3.3 Shape-IoU and Slide Loss

Bounding box regression loss considers three crucial geometric factors: the area of overlap, distance, and aspect ratio. By unifying the coordinates, IoU loss considers the overlap area, and GIoU loss builds on IoU loss but introduces the concept of the minimum enclosing box. DIoU loss proposed by Zheng et al. (2019) extends the traditional IoU loss by incorporating not only the overlap area between the predicted and ground truth (GT) bounding boxes but also their central points’ distance. Further improving upon DIoU, the CIoU loss was proposed to incorporate the geometric factor of the aspect ratio of the bounding box. Moreover, the SIoU method introduces a new loss term that considers the angular size of the line connecting the center points of the predicted and GT bounding boxes.

In summary, previous bounding box regression methods improve accuracy by adding geometric constraints to IoU. The YOLOv8n framework employs the Complete IoU (CIoU) loss function, which integrates supplementary components accounting for the aspect ratio and spatial alignment of predicted boxes, enhancing the stability of target box regression. This approach helps prevent issues such as divergence during training, which can occur with traditional IoU or GIoU losses.

**Figure 6:**
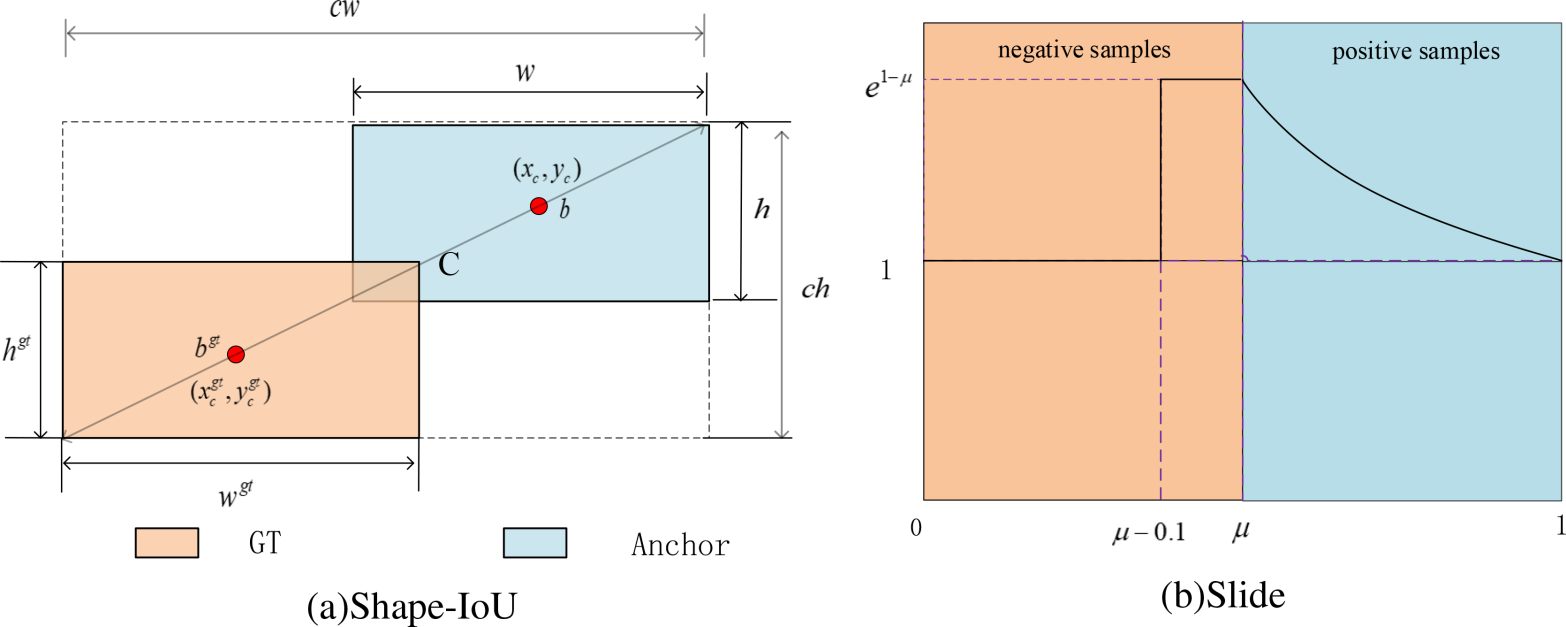
Localization and classification loss functions.

However, existing studies, including those using CIoU, DIoU, SIou, and GIoU overlook the impact of the bounding box’s shape and scale on the regression process, leading to suboptimal performance, particularly in scenarios where some scale or aspect ratio of objects varies significantly across the dataset. Addressing these factors could refine the bounding box prediction and improve model accuracy in diverse and dynamic real-world applications. Then, the CIoU loss used by YOLOv8n was replaced with the Shape-IoU loss function, which is shown in **Figure 6a**.

Where *ch* and *cw* are the height and width of the smallest rectangle that includes the two bounding boxes, *scale* is the scale factor. The terms *ww* and *hh* denote weighting coefficients along the horizontal and vertical axes, respectively, and are defined based on the geometry of the GT bounding box. These coefficients are derived from the aspect ratio of the GT bounding box and are used to modulate the loss function in accordance with the bounding box shape. Equation 27 illustrates the bounding box regression loss based on Shape-IoU.


(27) $$L_{Shape-IoU}=1-IoU+dis\text{tan }ce^{shape}+0.5\times {\text{Ω}}^{shape}$$

The variables involved above are calculated as Equations 28–34:


(28) $$IoU=\frac{\left|Box\cap^{}Box^{gt}\right|}{\left|Box\cup^{}Box^{gt}\right|}$$

(29) $$ww=\frac{2\times {\left(w^{gt}\right)}^{scale}}{{\left(w^{gt}\right)}^{scale}+{\left(h^{gt}\right)}^{scale}}$$

(30) $$hh=\frac{2\times {\left(h^{gt}\right)}^{scale}}{{\left(w^{gt}\right)}^{scale}+{\left(h^{gt}\right)}^{scale}}$$

(31) $$c^{2}={\left(cw^{2}+ch^{2}+eps\right)}^{alpha},eps=1e-7,alpha=1$$

(32) $$\begin{array}{c} distance^{shape}=hh\times {\left(x_{c}-x_{c}^{gt}\right)}^{2}/c^{2}+ww\times \\ {\left(yc-y_{c}^{gt}\right)}^{2}/c^{2} \end{array}$$

(33) $$\Omega^{shape}=\sum_{t=w,h}{\left(1-e^{-wt}\right)}^{\theta },\theta =4$$

(34) $$\{\begin{array}{l} \omega_{w}=hh\times \frac{\left|w-w^{gt}\right|}{\max\;(w,w^{gt})}\\ \omega_{h}=ww\times \frac{\left|h-h^{gt}\right|}{\max\;(h,h^{gt})} \end{array}$$

In classification evaluation, YOLOv8n utilizes binary cross entropy (BCE) loss. Due to the rice pests’ automatic detection stage, the original rice pest image dataset exhibits class imbalance, with an unequal distribution of positive and negative samples. Additionally, variations in pest target scales, image blurring due to equipment shaking during capture, and occlusions from leaves hinder effective detection. These challenges, coupled with the limitations of BCE loss make it difficult to differentiate between these samples. Therefore, Slide loss is employed as the classification loss, as depicted in **Figure 6b**.

Slide loss adaptively learns the threshold parameters *µ* for different samples. By assigning higher weights near *µ*, the loss of difficult-to-classify examples is increased, focusing more on complex and misclassified samples, and a weighting function is defined in Equation 35.


(35) $$f\left(x\right)=\{\begin{array}{ll} 1 & x\leq u-0.1\\ e^{1-u} & u<x<u-0.1\\ e^{\frac{1-x}{u}} & x\geq u \end{array}$$

### 3.4 Different object detection models and datasets

YOLOv3 (Redmon and Farhadi, 2018) incorporates the Darknet-53 backbone in conjunction with a Feature Pyramid Network (FPN), enabling the model to detect objects at three different scales: 13x13, 26x26, and 52x52. YOLOv5 (Kumar et al., 2023) switched to the PyTorch architecture and used the SPPF layer to improve detection efficiency. YOLOv6 (Li et al., 2022) combines the RepVGG and CSPStackRep modules to better express features based on a hybrid channel strategy. In addition, YOLOv8 (Sohan et al., 2024) has expanded multiple versions (n, s, m, l, x), which it used for various real-world tasks such as face detection and pose estimation. Recent advancements in the YOLO architecture demonstrate a clear trajectory toward optimized real-time object detection. YOLOv9’s innovation centered on its Programmable Gradient Information (PGI) mechanism, which effectively mitigates information degradation in deep networks while maintaining computational efficiency (Wang et al., 2024b). The subsequent YOLOv10 iteration introduces a hardware-aware neural architecture, significantly reducing latency without compromising detection fidelity (Wang et al., 2024a).

The study utilizes a composite rice pest dataset comprising both public and proprietary sources. Public datasets, including IP102 (Wu et al., 2019), Kaggle (Dataset Kaggle.com, 2022), UCI ML (Dataset archive.ics.uci.edu, 2019), and Mendeley (Dataset data.mendeley.com, 2022), are curated by governmental agencies, academic institutions, and research consortia. These repositories are characterized by extensive taxonomic diversity (encompassing multiple crop species) and large-scale annotated samples. Then, to rigorously assess model generalizability, this study employed 19000 annotated images from the IP102 (Wu et al., 2019) benchmark, a large-scale dataset designed for pest identification. IP102 spans 102 pest categories, exhibiting a long-tail distribution that reflects real-world ecological prevalence. It encompasses both soil-dwelling pests and crop-specific infestations (*e.g.*, rice, maize, and soybean), providing a robust data based for evaluating taxonomic and environmental variability.

### 3.5 Experimental settings

The experiment was conducted in a Python 3.8 environment on a 64-bit Ubuntu 20.04 operating system. The PyTorch framework version 1.11.1.1 was utilized, with a 16-core Intel ^®^ Xeon ^®^Platinum 8352*V* CPU and a 24*GB* RTX 4090 GPU for computation. *CUDA*11.3 was employed to accelerate operations. The relevant parameter settings used during model training are provided in **Table 3**. In addition, the model was trained for 100 epochs, with a learning rate of 0.01, a batch size of 16, an image normalization size of 640 × 640, a random seed of 0, an SGD optimizer, and a pre-trained model was loaded.

**Table 3:** Model training parameter settings.

Configuration	Parameter	Configuration	Parameter	Configuration	Parameter
epochs	100	lr0	1E-2	box_loss_gain	7.5
patience	50	Lrf	1E-2	close_mosaic	10
batch	16	momentum	0.937	cls_loss_gain	0.5
imagesize	640	weight_decay	0.0005	dfl_loss_gain	1.5
workers	8	warmup_epochs	3.0	amp	True
pretrained	True	warmup_momentum	1.5	fraction	1.0
optimizer	SGD	warmup_bias_lr	0.1	max_det	300
device	GPU	keypoint_obj_loss_gain	1.0	dropout	0.0
seed	0	Mosaic	1.0	mask_ratio	4

### 3.6 Model evaluation

This paper mainly analyzes and evaluates the model’s performance using precision (P), recall (R) rate, F1 value, and mAP.

Performance metrics (Padilla et al. (2020); Everingham et al. (2010)) are crucial for evaluating object detection algorithms like YOLOv8n, providing insights into how well the model detects and localizes objects. Precision measures the percentage of correctly detected objects (Equation 36), while Recall rate expresses the ratio of correctly predicted targets to actual targets(Equation 37). An ideal model would have both precision and recall near 100%. The F1 score considers precision and recall, maximizing both and achieving a balance (Equation 38).


(36) $$P\left(Precision\right)=\frac{TP}{TP+FP}$$

(37) $$R\left(\text{Re}call\right)=\frac{TP}{TP+FN}$$

(38) F1=2×(Precision×Recall)/(Precision+Recall)

True Positives (TP) represent the number of pest targets correctly predicted, and False Positives (FP) represent the number of non-pest targets predicted as pests. The recall rate quantifies the model’s ability to correctly identify instances of the target class, reflecting its sensitivity to true positive detections. Correspondingly, the False Negative (FN) metric denotes the number of pest instances that were present in the ground truth but were not detected by the model, indicating missed detections.

Mean Average Precision (mAP) is computed as the average of the individual Average Precision (AP) scores across all object categories, and serves as a principal metric for quantifying overall detection accuracy (Equation 39).


(39) $$mAP=\frac{\sum_{i=1}^{K}AP_{i}}{K}$$

(40) $$AP_{i}=\int_{0}^{1}p_{i}\left(R_{i}\right)d\left(R_{i}\right)$$

(41) $$mAP_{0.5-0.95}=\frac{mAP_{0.5}+mAP_{0.55}+\cdots +mAP_{0.95}}{10}$$

where *K* denotes categories, and *AP_i_* indicates the average accuracy of the *i* − *th* category. The *AP* (Equation 40) metric is derived by computing the area under the precision-recall curve, and *mAP*_0_._5_ refers to the *mAP* calculated at an intersection over the union (IoU) threshold of 0.5, indicating that detection is considered correct if the IoU exceeds 0.5. *mAP*_0_._5−0_._95_ (Equation 41) metric provides a more comprehensive evaluation of the model’s detection performance by averaging the mAP across multiple IoU thresholds, typically ranging from 0.5 to 0.95 in increments of 0.05.

## 4 Results

### 4.1 Model training results

**Figure 7:**
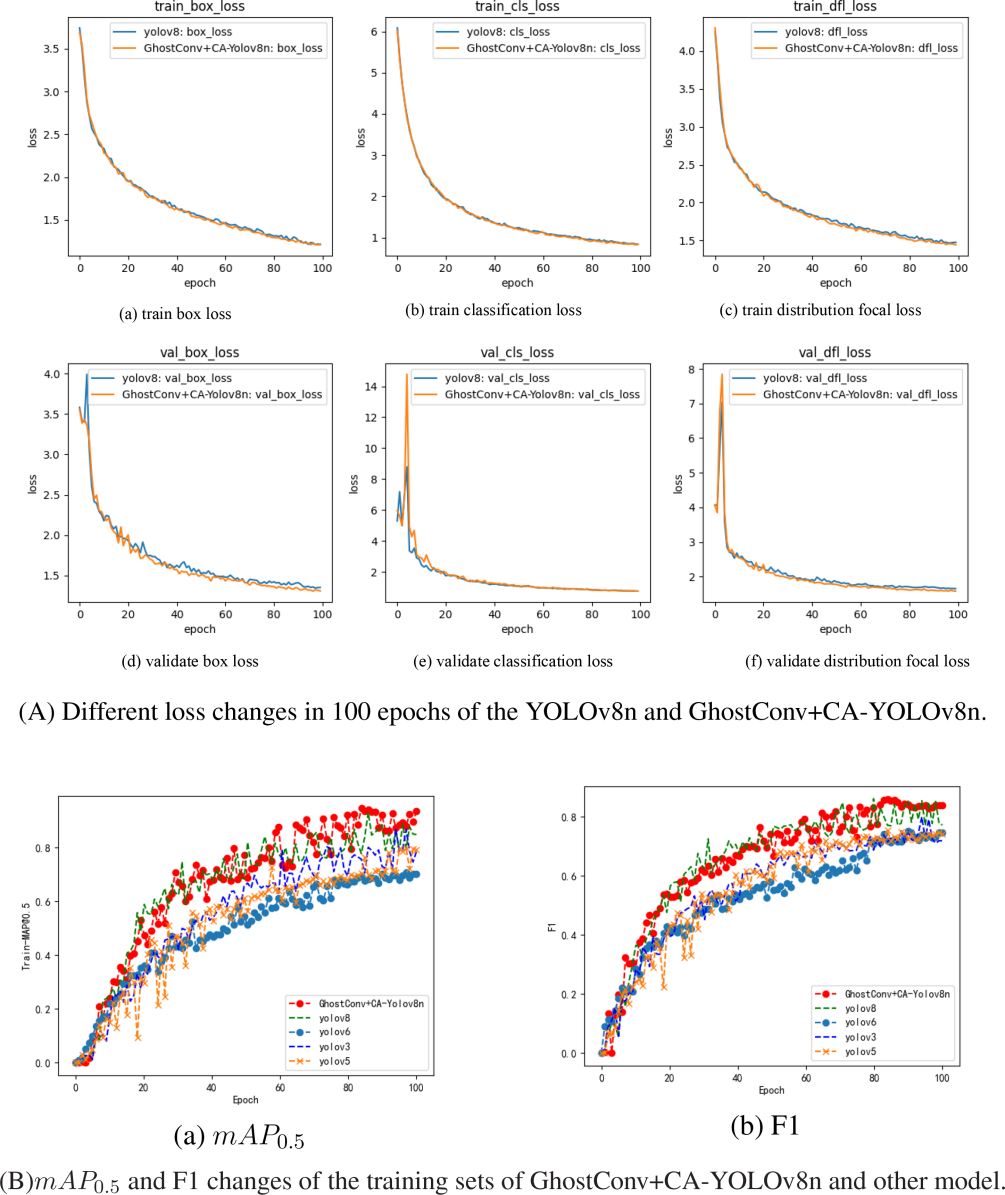
The loss and evaluation results of the model training process.

YOLOv8n’s losses include Box loss, CLS loss, and DFL loss. Box loss (bounding box loss) helps improve object localization accuracy by minimizing the difference between predicted and ground-truth bounding boxes. Cls loss (Classification loss) enhances YOLOv8n’s ability to classify detected objects correctly. DFL Lin et al. (2017) addresses class imbalance and hard-to-detect objects, and it achieves this by placing greater emphasis on complex or difficult examples, thereby enhancing the detection of rare or small objects. In the article, the loss change results of 100 epochs of model training are shown in **Figure 7**.

From **Figure 7A**, The YOLOv8n algorithm attains optimal detection accuracy within 60 training epochs, and GhostConv+CA-YOLOv8n model exhibited box and classification losses of 1.4511 and 1.123 on the training set at 60 epochs, with corresponding validation losses of 1.4324 and 0.96125. Relative to the baseline YOLOv8n model, these values reflect reductions of 0.0177 and 0.0024 on the training set, and 0.0468 and 0.02443 on the validation set, respectively. These improvements suggest that incorporating GhostConv and Context Aggregation enhances convergence efficiency and accelerates regression during training. Moreover, the *mAP*_0_._5_ and F1 score curve of the YOLOv8, YOLOv6, YOLOv5, YOLOv3 and GhostConv+CA-YOLOv8n, as shown in **Figure 7B**. The proposed algorithm’s *mAP*_0_._5_ curve is higher than the MAP curves of other YOLO algorithms.

### 4.2 Ablation experiment

Ablation studies were conducted on the RicePest15, **Table 4** shows the incremental improvements in detection performance achieved by each strategy.

**Table 4:** Ablation experiment.

Algorithm	Precision	Recall	F1	*mAP*_0.5_	*mAP*_0.5−0.95_	Parameters	Gradients
YOLOv8n	0.86302	0.70668	0.77706	0.84994	0.5331	3013773	3013757
+GhostConv	0.76668	0.65043	0.70378	0.72645	0.46859	2823933	2823917
+ContextAggregation	0.86308	0.73254	0.79247	0.80892	0.55062	3163155	3163139
+GhostConv+ContextAggregation(neck)	0.77928	0.90889	0.83911	0.92388	0.54462	2973315	2973299

Substituting the conventional convolutional with GhostConv in YOLOv8n’s backbone architecture resulted in a reduction of model complexity, decreasing the number of parameters and gradients by 189,840. Integrating Context Aggregation attention into the prediction head improved feature extraction, refining the model’s focus on crucial features and improving *Precision*, *Recall*, *F*1 score, and *mAP*_0.5−0.95_, by 0.006%, 2.586%, 1.541%, and 1.752%, respectively. Combining GhostConv and ContextAggregation led to a 7% improvement in *mAP*_0.5_, and a reduction of 40,458 parameters and gradients. It highlights the effectiveness of the YOLOv8n+GhostConv+ ContextAggregation (neck) in optimizing model size and accuracy.

Furthermore, the captured pest images exhibit significant variability due to multi-scale target distributions, lighting inconsistencies, and motion blur, which collectively pose challenges for the accurate detection of occluded pests. To address these complexities, the integration of attention mechanisms into the backbone or neck of the network enhances the model’s capacity to capture salient structural and contextual features, thereby improving its ability to discriminate between target pests and background noise. This paper uses precision, recall, F1 value, *mAP*_0.5_, *mAP*_0.5−0.95_, parameter and gradient as evaluation indicators and conducts comparative tests by adding multiple attention mechanisms in different parts, the results as shown in **Table 5a**.

**Table 5:** The experimental results of yolov8n under different attention mechanism strategies.

(a) Comparison of adding attention mechanism to backbone and neck in YOLOv8n									
Algorithm	backbone	neck	Precision	Recall	F1	*mAP*_0.5_	*mAP*_0.5−0.95_	Parameters	Gradients
+SEAttention	✓		0.67949	0.66818	0.67378	0.7301	0.42054	3021965	3021949
+GhostConv+ SEAttention	✓		0.79629	0.68519	0.736574	0.75569	0.43619	2832125	2832109
+GhostConv+ SEAttention		✓	0.9015	0.55695	0.68852	0.77592	0.42831	2824701	2824685
(b) Compared adding different attention mechanisms to the neck using GhostConv in YOLOv8n’s backbone									
Algorithm		neck	Precision	Recall	F1	*mAP*_0.5_	*mAP*_0.5−0.95_	Parameters	Gradients
+GhostConv+Global- Context Block		✓	0.80373	0.71382	0.75611	0.78932	0.42547	5241503	5241487
+GhostConv+Non- Local Block		✓	0.86385	0.64065	0.73569	0.74893	0.40661	5529469	5529453
+GhostConv+ SKAttention		✓	0.67292	0.7945	0.72867	0.85887	0.45572	12148413	12148397
+GhostConv+BoT3		✓	0.89515	0.7251	0.8012	0.82979	0.54226	5468413	5468397
+GhostConv+ ShuffleAttention		✓	0.67949	0.66818	0.67379	0.7301	0.42054	2824077	2824061
+GhostConv+ ECAAttention		✓	0.86788	0.63274	0.73189	0.73277	0.43846	2823939	2823923
+GhostConv+ EffectiveSE		✓	0.68048	0.7802	0.726936	0.80774	0.44522	2906237	2906221
+GhostConv+MHSA		✓	0.8136	0.70579	0.75587	0.78298	0.42662	5789309	5789293
+GhostConv+Context-Aggregation		✓	0.77928	.90889	0.83911	0.92388	0.54462	2973315	2973299
(c) Ablation results of adding multiple attention mechanisms to the backbone and neck in YOLOv8n.									
Algorithm	neck	backbone	Precision	Recall	F1	*mAP*_0.5_	*mAP*_0.5−0.95_	Parameters	Gradients
+ContextAggregation+SEAttention	✓	✓	0.73317	0.88772	0.803077	0.88874	0.51551	5194387	5194371
+GhostConv+ContextAggregation+SEAttention	✓	✓	0.77719	0.84778	0.810952	0.85862	0.49923	5004547	5004531
+ContextAggregation+ MHSA	✓	✓	0.7627	0.69166	0.725445	0.77163	0.39615	5391251	5391235
+GhostConv+ContextAggregation+MHSA	✓	✓	0.7978	0.68061	0.73456	0.75133	0.50689	5201411	5201395
+GhostConv+ContextAggregation	✓		0.77928	0.90889	0.83911	0.92388	0.54462	2973315	2973299

As demonstrated in **Table 5a**, incorporating GhostConv into YOLOv8n’s backbone, along with the integration of attention mechanisms into both the backbone and neck components. It is found that the above modifications not only reduced the number of model parameters and computational gradients but also yielded notable performance improvements, with improvements of 4.582% (backbone) and 2.559% (neck) in *mAP*_0_._5_, respectively.

Ablation studies were conducted with different attention mechanisms applied to the neck, as presented in **Table 5b**. The results show that adding the context aggregation mechanism to the neck network can improve the *mAP*_0_._5_ value of the YOLOv8n model to 92.388%, which is 9.4% higher than GhostConv+ BoT3 (82.979%) and 17.50% higher than GhostConv+ Non-Local Block(74.893%). Notably, it achieves the highest F1 score (83.911%), surpassing GhostConv+ BoT3 (80.12%) by 3.80% and GhostConv+ ShuffleAttention(67.379%) by 16.532%. Its mAP0.5-0.95 is 54.462%, which is also better than all other models. Although its accuracy (77.928%) is slightly lower than some alternatives, its recall (90.889%) is the highest, exceeding GhostConv+ BoT3 (72.51%) by 18.38% and GhostConv+ ECAAttention (63.274%) by 27.615%. In addition, CA-YOLOv8n contains only 2,973,315 parameters, which is still much lighter than models such as GhostConv+ SKAttention (12,148,413 parameters) and GhostConv+ BoT3 (5,468,413 parameters), while reducing computational complexity while maintaining excellent accuracy.

A comprehensive attention mechanism is adopted by incorporating an attention block into YOLOv8n’s neck and backbone networks. The context-aggregation module is added to the neck, while SEAttention and MHSA are the backbone network. As presented in **Table 5c**, the results suggest that adding multiple attention mechanisms does not necessarily improve performance. This outcome may be attributed to the increased likelihood of training deviations when multiple attention mechanisms are simultaneously employed, causing a bias in the focused features and ultimately leading to suboptimal model training.

According to **Table 5c**, the YOLOv8n+ GhostConv+ ContextAggregation model achieves a *mAP*_0_._5_ of 92.388%, outperforming the YOLOv8n+ ContextAggregation+ MHSA model (77.163%) by 15.225% and exceeding the weakest-performing variant (75.133%) by 17.255%. Additionally, it demonstrates a 3.514% improvement over the SEAttention-enhanced model (88.874%), highlighting its superior detection capabilities. The YOLOv8n+ GhostConv+ ContextAggregation model maintains a lightweight architecture with only 2.84M parameters, representing a 42.62% reduction in model complexity compared to the YOLOv8n+ ContextAggregation+ SEAttention model (4.95M) while achieving superior detection performance.

This study utilized different loss functions to optimize the model, which is represented as +MPDIoU+BCE, +GIoU+BCE, +WIoU+BCE, and +ShapeIoU+Slide (**Table 6**).

**Table 6:** Comparison of model effects under different loss functions.

Algorithm	Precision	Recall	F1	*mAP*_0.5_	*mAP*_05-0.95_	Parameters	Gradients
YOLOv8n+GhostConv+ContextAggregation (CIoU+BCE)	0,77928	0.90889	0.83911	0.92388	0.54462	2973315	2973299
+MPDIoU+BCE	0.84973	0.78267	0.814823	0.83924	0.45046	2973315	2973299
+GIoU+BCE	0.71777	0.76397	0.74015	0.78277	0.45977	2973315	2973299
+WIoU+BCE	0.80973	0.71247	0.75799	0.77713	0.41711	2973315	2973299
+ShapeIoU+Slide (GhostConv+CA-YOLOv8n)	0.89959	0.82258	0.859363	0.94527	0.55579	2973315	2973299

The optimized loss function +ShapeIoU+Slide is the most significant. Specifically, the GhostConv+CAYOLOv8n model incorporates the ShapeIoU+Slide loss function to optimize the original CIoU+BCE loss, attaining the highest *mAP*_0_._5_ of 94.527%, and achieve precision increase of 12%(77.928% to 89.959%) and a 2% improvement in the F1 score (83.911% to 85.936%) compared to the YOLOv8n+ GhostConv+ ContextAggregation(CIoU+BCE) model.

### 4.3 Comparison of different object detection methods

To validate the benefits of our approach, some classic models, including the YOLO series (YOLOv3 through YOLOv10) (see **Table 7a**), SSD, and FasterRCNN, was compared on the Ricepest15 dataset (see **Table 7b**). SSD utilized VGG16, ResNet50, and MobileNetV2 as backbone networks, while FasterRCNN employed VGG16 and ResNet50 (101, 152).

**Table 7:** The comparison results of different target detection methods.

(a) Comparison results of YOLO series models								
Algorithm	Precision	Recall	F1	*mAP*_0.5_	*mAP*_0.5−0.95_	Parameters		Gradients
YOLOv3	0.78012	0.66557	0.71831	0.78519	0.45815	103704029		103704013
YOLOv3+tiny	0.79975	0.78118	0.79036	0.81089	0.49266	12139838		12139822
YOLOv5	0.83948	0.66124	0.73978	0.74564	0.36464	2511389		2511373
YOLOv5s	0.85732	0.72964	0.78834	0.81336	0.46122	9127997		9127981
YOLOv5-fpn	0.79496	0.5785	0.66967	0.62204	0.27811	83809277		83809261
YOLOv5-p6	0.8592	0.64922	0.74000	0.79245	0.41758	4137516		4137500
YOLOv6	0.8593	0.65834	0.74551	0.70192	0.38311	4239629		4239613
YOLOv7	0.90049	0.8618	0.88072	0.92082	0.54989	44231325		44231309
YOLOv7x	0.82989	0.558	0.667313	0.78717	0.39219	44231325		44231309
YOLOv8n-p2	0.79094	0.71206	0.74943	0.75452	0.46748	2928540		2928524
YOLOv8n-p6	0.83912	0.88417	0.86106	0.91316	0.54868	4786972		4786956
YOLOv8n- MobileNetv3	0.85575	0.75551	0.80252	0.82352	0.52937	5976985		5976969
Yolov9	0.91195	0.77293	0.836705	0.8686	0.49182	60829562		60829530
Yolov10	0.75808	0.78669	0.77212	0.82335	0.50957	20474154		20474138
GhostConv+CAYOLOv8n *☆*	0.89959	0.82258	0.859363	0.94527	0.55579	2973315		2973299
(b) Comparison results of SSD and FasterRCNN models.								
Algorithm		*mAP*_0.5_			*mAP*_0.5−0.95_			
FasterRCNN + vgg16		76.83%			–			
FasterRCNN + resnet50		89.01%			–			
FasterRCNN + resnet101		86.16%			–			
FasterRCNN + resnet152		89.53%			–			
SSD+vgg16		90.60%			55.40%			
SSD + mobilenetv2		67.10%			30.00%			
SSD+ resnet50		72.80%			37.10%			
GhostConv+CA-YOLOv8n *☆*		94.527%			55.579%			
(c) Ablation experiment results on IP102 test set.								
Algorithm	Precision	Recall	F1	*mAP*_0.5_	*mAP*_0.5−0.95_	parameters	gradients	
YOLOv8n	0.46203	0.50064	0.480561	0.49381	0.31396	3013773	3013757	
GhostConv+CAYOLOv8n	0.51655	0.53471	0.525473	0.49733	0.31399	2973315	2973299	

According to the results in **Tables 7a, b**, the GhostConv+CA-YOLOv8n model has significant advantages over other YOLO variants, outperforming YOLOv3, YOLOv5, YOLOv7, YOLOv8n in *mAP*_0_._5_ (94.527%) and *mAP*_0.5−0.95_ (55.579%). Although GhostConv+CA-YOLOv8n is 1.236% lower than the highest YOLOv9 model in precision (89.959% vs. 91.195%), it is higher than YOLOv9 in recall and F1 by 4.965% (82.258% vs. 77.293%) and 2.2658% (85.9363% vs. 83.6705%). In addition, compared with the better yolov7 model, our model is 0.09% (89.959% vs. 90.049%), 3.9% (82.258% vs. 86.18%), and 2.1357% (85.9363% vs. 88.072%) lower than yolov7 in precision, recall, and F1 value, but *mAP*_0_._5_ (94.527% vs. 92.082%) and *mAP*_0.5−0.95_ (55.579% vs. 54.989%) are 2.445% and 0.59% higher than yolov7. The model parameters and gradient number are reduced by 14.88 times compared with yolov7. Therefore, the GhostConv+CA-YOLOv8n model has the highest average detection accuracy while maintaining a lightweight structure with fewer parameters and gradients.

In addition, GhostConv+CA-YOLOv8n model performs well compared to other mature models, including FasterRCNN and SSD, achieving *mAP*_0_._5_ of 94.53% and *mAP*_0.5−0.95_ of 55.58%, which is 3.93% (94.53% vs. 90.60%) and 0.18% (55.58% vs. 55.40%) higher than the best SSD+vgg16 among all the compared models. And the superior FasterRCNN + resnet152 model is 5% higher in *mAP*_0_._5_ (94.53% vs. 89.53%). It highlights its ability to detect pests consistently and accurately, which is critical for agricultural applications.

### 4.4 Model comparison of public datasets

The public datasets for plant diseases and pests detection are PlantVillage, Rice Leaf Diseases Data Set, Image Database for Plant Disease Symptoms (PDDB) Garcia Arnal Barbedo et al. (2018), New Plant Diseases Dataset (Augmented), DAGS Brahimi et al. (2018), Northern Leaf Blight (NLB) dataset Stewart et al. (2019), IP102 Wu et al. (2019), tomato pest images Huang and Chuang (2020), apple leaf disease Thapa et al. (2020) and PlantDoc Singh et al. (2020). However, these datasets were primarily constructed under controlled or laboratory conditions, limiting the generalizability of the trained models, which often exhibit suboptimal performance when applied to real-world imagery.

IP102 dataset comprises 102 categories, including crops, fruits, vegetables, and various pests relevant to agriculture. The training metrics of the YOLOv8n and GhostConv+CA-YOLOv8n algorithms on the IP102 (containing 19,000 images) dataset are presented in **Table 7c**.

Quantitative evaluations reveal statistically significant improvements across all metrics: GhostConv+CA-YOLOv8n attains 51.655% precision (Δ+ 5.452% vs. baseline), 53.471% recall (Δ+3.407% vs. baseline), 52.5473% F1(Δ+ 4.4912% vs. baseline), and 49.733% *mAP*_0_._5_ (Δ+ 0.352% vs. baseline), outperforming contemporary models in cross-dataset validation on IP102.

### 4.5 Visualization of detection effects

Under the natural growth conditions of rice, the background of pests in the field is complex. For example, some pests appear not only on rice but also on other crops with specific mobility. Secondly, crop leaves will block the pests, making it challenging to extract complete pest features, and the target detection performance is subpar. The resolution of the captured images varies, making detecting tiny target pests more difficult.

**Figure 8:**
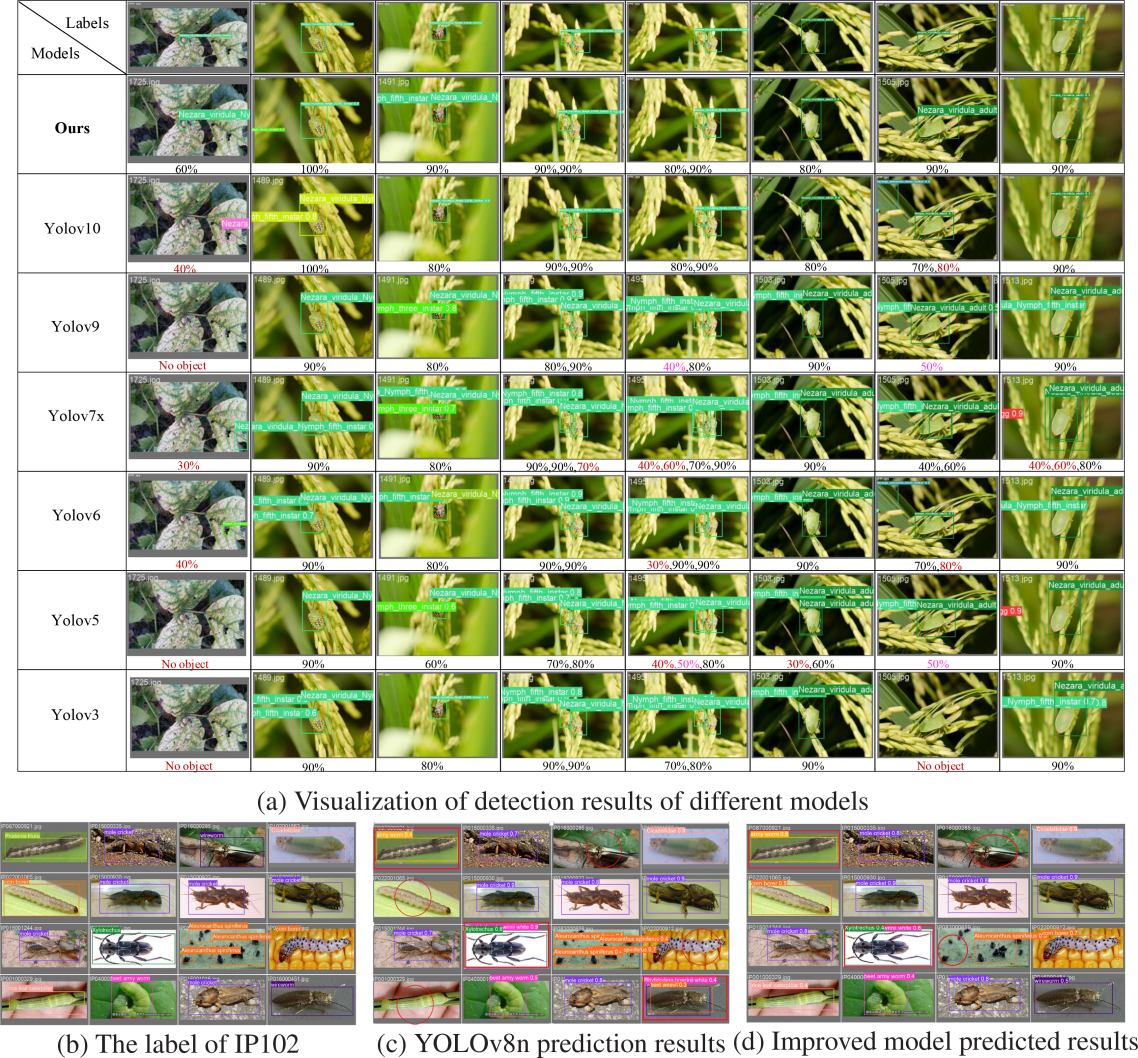
Visualization of model prediction results for Ricepest15 and IP102(test set).

This study employs seven algorithms from the YOLO series to detect randomly selected rice pest images from the Ricepest15 test dataset. The visualization results are presented in **Figure 8a**. Among them, the dark red numbers indicate the confidence corresponding to the false detection objects of the seven models. Bright red (pink) indicates that the confidence of the model prediction is less than or equal to 50%. The visualization output provides an intuitive comparison of the proposed algorithm with other compared algorithms regarding categories and confidence.

The GhostConv-CA-YOLOv8n model demonstrates superior accuracy in predicting bounding boxes, exhibiting precise alignment with the target object (labeled data), as shown in the second row of **Figure 8a**. In comparison, models such as YOLOv10, YOLOv7x, YOLOv6, YOLOv5, and YOLOv3 exhibit false positives and false negatives. The GhostConv-CA-YOLOv8n model consistently achieves recognition accuracy ranging from 80% to 100% across various test cases. Notably, the visualization results reveal that some models, including YOLOv9 and YOLOv10, achieve high accuracy in certain instances but demonstrate inconsistent performance in more complex scenarios, such as inter-target occlusion (fifth column of the fourth row), varying cropped backgrounds (first column of the third and fourth rows), and lowcontrast environments (seventh column of the third and fourth rows). In contrast, GhostConv-CA-YOLOv8n maintains high detection confidence and an improved detection rate across all test conditions.

The study conducted a comparative experiment against the YOLOv8n baseline model using the publicly available IP102 dataset. A batch processing approach was employed, where sixteen images were randomly selected for prediction. Both models processed the images sequentially, and their respective predictions were concatenated. Finally, the prediction results from both models were compared against the ground truth labels in the test set, yielding the results presented in **Figures 8c, d**.

The circles and rectangles in **Figures 8c, d** highlight a comparison of the original and improved models’ missed detection and false detection results. In **Figure 8b**, the target in row (1) is darker than the background, and the small target features in row (3) are not prominent. GhostConv+CA-YOLOv8n model has some missed detections and false detections. However, the number of false detections (row (1) column (1)) and missed detections (row (1) column (3) and row (3) column (3)) of the GhostConv+CA-YOLOv8n model are lower than YOLOv8n (row (1) column (1), row (3) column (2), and row (4) column (4) are three false detections; row (1) column (3), row (2) column (1) and row (4) column (1) are three missed detections).

The comparative analysis of the YOLOv8n baseline model, the GhostConv+CA-YOLOv8n model, and the ground truth labels, the GhostConv+CA-YOLOv8n model showed a higher confidence score and higher classification accuracy, effectively reducing the misclassification in the baseline model. Notably, it accurately identifies species such as Rice leaf caterpillar, Corn borer, and Wireworm, demonstrating closer alignment with the ground truth annotations. Furthermore, the GhostConv+CA-YOLOv8n model exhibits enhanced object localization capabilities, with more precise bounding boxes (*e.g.*, beet armyworm) and better discrimination of visually similar species (*e.g.*, beet weevil and wireworm in the fourth row and fourth column). These results manifest the potential of GhostConv+CA-YOLOv8n to improve the accuracy and reliability of pest detection, providing a valuable tool for agricultural pest monitoring and management.

In summary, as demonstrated in **Figure 8**, the proposed model exhibits the fewest false positives and missed positives, and demonstrates higher confidence in category predictions, highlighting the effectiveness and transferability of the GhostConv+CA-YOLOv8n.

## 5 Discussion

This paper proposed that the GhostConv+CA-YOLOv8n model has successfully diagnosed rice pests. The performance superiority stems from three synergistic mechanisms: (a) The GhostConv’s parameter redistribution strategy maximizes feature representational capacity per computational unit (**Figure 4**); (b) The CA-block’s non-local attention gates amplify discriminative features in occluded scenarios (**Figure 8**); (c) Shape-IoU and Slide loss prove advantageous in accurately defining the pest boundaries and addressing the imbalance between easy (non-occluded) and complex (occluded) samples(*mAP*_0_._5_ ↑ 9.533%). The specific discussion is as follows:

### 5.1 Comparison with traditional data annotation tools

This paper uses the latest cutting-edge model–SAM (Kirillov et al., 2023), which can effectively locate the boundaries of rice pests and convert them into the format of YOLOv8n (Wang et al., 2024c) model training, thereby improving the efficiency of manual annotation of rice pests. Compared with the traditional Labelme annotation method, SAM usually adopts interactive annotation as a general large model obtained by training with massive data (Zhang et al., 2024). People only need to click the pest area with the mouse (click the prompt word) to mask similar pest areas and select pests in batches, without selecting pests one by one like Labelme, saving a lot of human resources.

### 5.2 Performance evaluation of the GhostConv+CA-YOLOv8n model

A detailed comparison of the GhostConv+CA-YOLOv8n model against other well-known algorithms, such as YOLOv3, YOLOv5, YOLOv7, and YOLOv8n’s variants, highlights the strengths and efficiency of the proposed model.

• Precision and Recall

GhostConv+CA-YOLOv8n achieved 89.959% precision, demonstrates impressive performance in rice pest detection, which is 11.947% higher than YOLOv3 (89.959% vs. 78.012%) and 4.384% higher than YOLOv8n-MobileNetv3 (89.959% vs. 85.575%). This indicates that GhostConv+CAYOLOv8n is better at reducing false positives in its predictions, making it a more reliable model for rice pest detection. Furthermore, its Recall of 82.258% is 15.701% higher than YOLOv3 (82.258% vs. 66.557%) and 6.71% higher than YOLOv8n-MobileNetv3 (82.258% vs. 75.551%). A higher recall implies that the model is more effective at detecting true positive instances, even in challenging conditions.

• F1 Score and mAP Performance

The F1 Score, a critical metric for balanced performance, is 85.936% for GhostConv+CA-YOLOv8n, showing a substantial improvement of 19.65% over YOLOv3 (71.831%) and 7.13% over YOLOv8n-MobileNetv3 (80.252%). This improvement reflects the model’s superior capability to balance both precision and recall.

*mAP*_0_._5_ metric for GhostConv+CA-YOLOv8n reaches 94.53%, marking an exceptional increase of 20.37% over YOLOv3 (Redmon and Farhadi, 2018) (78.519%) and 3.51% over YOLOv8n-p6 (He et al., 2025) (91.316%). This significant enhancement in *mAP* demonstrates the model’s high accuracy in pest detection, especially when evaluating the performance at a threshold of 0.5 IoU (Intersection over Union). The model can detect pests of diverse conditions, and its *mAP*_0_._5−0_._95_ value of 55.579%, which is 6.33% higher than YOLOv3 (45.815%) and 1.28% higher than YOLOv8n-p6 (54.868%), highlighting the GhostConv+CA-YOLOv8n model’s improved performance in multiple evaluation scenarios.

• Parameter and Gradient Comparison GhostConv+CA-YOLOv8n model is a lightweight architecture. With only 2,973,315 parameters and 2,973,299 gradients, it dramatically reduces the computational load compared to other models, such as YOLOv3 (103,704,029 parameters and 103,704,013 gradients) and YOLOv5-fpn (Zhang et al., 2017) (83,809,277 parameters and 83,809,261 gradients). By replacing the standard convolution in YOLOv8n’s backbone with GhostConv, more than 180,000 parameters and gradients were reduced compared with base YOLOv8n. This reduction in parameters and gradients makes GhostConv+CA-YOLOv8n more efficient in memory usage and computational resources. Moreover, compared to YOLOv7x (T.P. et al., 2025) (44,231,325 parameters and 44,231,309), the GhostConv+CA-YOLOv8n model is approximately 67.22% smaller in size while still outperforming it in mAP and detection accuracy.

• Model Efficiency

Despite the substantial improvements in detection performance, the GhostConv+CA-YOLOv8n model remains highly efficient, requiring fewer resources than many other YOLO variants, including YOLOv7 (44,231,325 parameters), YOLOv5-p6 (4,137,516 parameters), and YOLOv5s (Wang and He, 2021)(9,127,997 parameters). The reduction in parameters, alongside the higher detection accuracy, through visualization analysis of the public IP102 dataset (**Figures 8b–d**) reveals that GhostConv+CA-YOLOv8n rarely missed or false detections in the images of pests and diseases. It indicates that the GhostConv+CA-YOLOv8n model strikes a balance between performance and computational efficiency, making it an attractive choice for deployment in practical applications where computational resources may be constrained.

### 5.3 Comparison with non-YOLO algorithms

GhostConv+CA-YOLOv8n achieves an impressive 94.53% in *mAP*_0_._5_, outperforming FasterRCNN with resnet152 (which achieved 89.53%) by 5.00%. This demonstrates that the GhostConv+CAYOLOv8n model provides significantly better performance in detecting pests at a relatively lower threshold, indicating a higher detection rate of true positives.

SSD with VGG16 (Liu et al., 2016) comes close with a *mAP*_0_._5_ of 90.60% but still lags behind GhostConv+CA-YOLOv8n by 3.93%. SSD with mobilenetv2 (Chiu et al., 2020) and resnet50 (Liu et al., 2019) show considerably lower performance, with *mAP*_0_._5_ of 67.10% and 72.80%, respectively. These models demonstrate the challenges of detecting small or occluded pests due to their lower precision and recall compared to the proposed model.

GhostConv+CA-YOLOv8n model shows a *mAP*_0_._5−0_._95_ score of 55.579%, which is higher than SSD with VGG16 (55.40%) and considerably better than SSD with mobilenetv2 (30%) and resnet50 (37.10%). Notably, FasterRCNN (Ren et al., 2015) models, including FasterRCNN with resnet50 (Tahir et al., 2021) (null) and FasterRCNN with resnet152 (Lee and Jo, 2020) (null), do not report a *mAP*_0_._5−0_._95_, limiting their comparability for this more stringent evaluation. However, the fact that GhostConv+CA-YOLOv8n maintains a robust *mAP*_0_._5−0_._95_ score signifies its consistency and ability to accurately detect pests over a range of IoU thresholds.

## 6 Conclusions

The occurrence of insect pests represents a substantial threat to rice production, often necessitating extensive pesticide application, which in turn contributes to ecological degradation. Therefore, the ability to rapidly and accurately detect rice pests is essential for both agricultural productivity and environmental sustainability.

GhostConv+CA-YOLOv8n enables efficient pest detection in complex environments and facilitates deployment on edge devices with limited computational resources. By incorporating Context Aggregation, the model effectively captures key feature map information, enhancing detection accuracy. Additionally, the GhostConv module optimizes the YOLOv8n backbone, reducing computational complexity while maintaining performance. Further improvements are achieved through the integration of Shape-IoU and Slide loss, which enhance global feature extraction and mitigate overfitting.

The GhostConv+CA-YOLOv8n outperforms conventional YOLO models (YOLOv3, YOLOv4, YOLOv5), as well as Faster R-CNN and SSD, achieving recognition precision of 89.959%, recall rate of 82.258%, *mAP*_0_._5_ of 94.527%, with a lightweight architecture of 2,973,315 parameters and 2,973,299 (2.84M) gradients. The above results highlight the accurate and efficient pest detection performance of the GhostConv+CA-YOLOv8n model, and the proposed framework enables real-time precision entomology in agronomic practice, providing: (1) Early detection of invasive species through morphological fingerprinting; (2) Pesticide reduction via threshold-based infestation alerts; (3) Ecological modeling with spatiotemporal pest distribution heatmaps, advancing sustainable rice cultivation paradigms.

Although the GhostConv+CA-YOLOv8n model has a high recognition rate when the characteristics of rice pests (occluded or less) are not prominent, it has broad application prospects. However, there are still certain limitations for further research and improvement: In large-scale rice cultivation under natural field conditions, real-time monitoring of pest activity necessitates the deployment of lightweight detection models on aerial platforms. By embedding the model within unmanned aerial vehicles (UAVs), and leveraging multispectral data acquired through onboard sensors, it becomes feasible to achieve efficient and continuous surveillance of rice pests *in situ*. Moreover, when rice leaves completely block the pests, or the pests are on the back of the rice leaves, or the pests migrate to other crops or hide under the land, this will reduce the effectiveness of GhostConv+CA-YOLOv8n.

## Data Availability

The raw data supporting the conclusions of this article will be made available by the authors, without undue reservation.
